# Estimating Lifetime Risk of Autosomal Recessive Kidney Diseases Using Population-Based Genotypic Data

**DOI:** 10.1016/j.ekir.2025.04.036

**Published:** 2025-04-21

**Authors:** Matthias Christoph Braunisch, Clara M. Großewinkelmann, Martin Menke, Nora Hannane, Riccardo Berutti, Jasmina Ćomić, Roman Günthner, Lutz Renders, Christoph Schmaderer, Uwe Heemann, Korbinian M. Riedhammer, Matias Wagner, Julia Hoefele

**Affiliations:** 1 Department of Nephrology, Klinikum rechts der Isar, Technical University of Munich, TUM School of Medicine and Health, Munich, Germany; 2 Institute of Human Genetics, Klinikum rechts der Isar, Technical University of Munich, TUM School of Medicine and Health, Munich, Germany; 3 Division of Nephrology, Department of Pediatrics, Boston Children's Hospital, Harvard Medical School, Boston, Massachusetts, USA; 4 Institute of Neurogenomics, Helmholtz Zentrum München, Neuherberg, Germany; 5 Institute of Human Genetics, University Hospital, Ludwig-Maximilians University, Munich, Germany

**Keywords:** autosomal recessive kidney disease, lifetime risk, monogenic kidney disease, population genetics, prevalence

## Abstract

**Introduction:**

Monogenic kidney diseases, though rare, exhibit a wide spectrum of clinical manifestations. The clinical and genetic diversity and potential biases in patient referrals and identification present challenges in accurately estimating prevalences based solely on phenotype. Our aim was to determine the calculated lifetime risk associated with autosomal recessive kidney diseases (ARKDs) using population-based genotype data.

**Methods:**

We conducted a comprehensive literature review to compile a list of 149 genes associated with ARKDs, including 31 glomerulopathies, 16 tubulopathies, 87 ciliopathies, and 15 congenital anomalies of the kidney and urinary tract (CAKUT). Disease-causing variants were collected from ClinVar, HGMD, LOVD, and our in-house database and evaluated for inclusion. Minor allele frequencies of 12,912 variants were then obtained from the Genome Aggregation Database (gnomAD) and the in-house database to estimate the lifetime risk.

**Results:**

The combined estimated lifetime risk was 27.49 per 100,000 (19.35–39.65) based on the European gnomAD dataset. The 3 disorders with the highest lifetime risk (>1.5 per 100,000), accounted for 24% of the overall lifetime risk and were caused by *PKHD1* (autosomal recessive polycystic kidney disease), *SLC12A3* (Gitelman syndrome), and *COL4A3* (Alport syndrome) variants. Extrapolating to all modes of inheritance, the overall lifetime risk for monogenic kidney disease ranged from 1 in 611 to 1 in 498.

**Conclusion:**

This study offers a comprehensive population-genetic assessment of the lifetime risk associated with ARKDs focusing on European populations, shedding light on previously underestimated prevalences and diagnostic probabilities. Consequently, these findings provide crucial insights for optimizing resource allocation towards therapy development, enhancing public health strategies, and guiding future biomedical research endeavors.

## Graphical abstract

**Figure:**
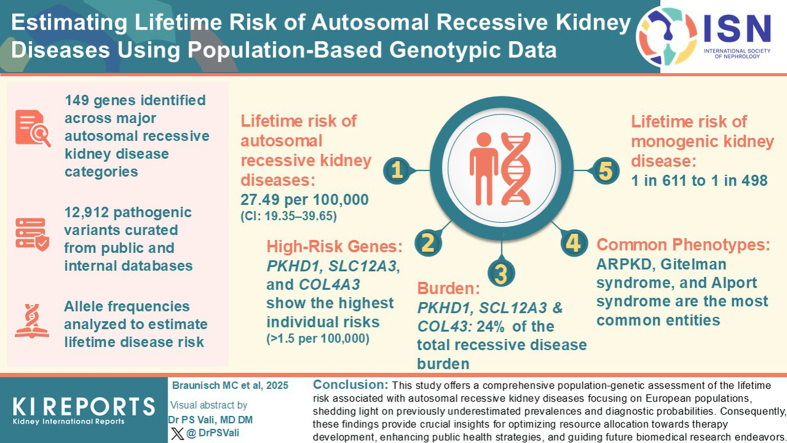

Monogenic kidney diseases comprise a group of clinically and genetically heterogeneous disorders.^1^, ^2^, ^3^ The estimated global prevalence of chronic kidney disease (CKD) is 11% to 13%.^4^ In Europe, CKD stages 3 to 5 have a prevalence of 12%.^4^ A monogenic disease-causing (likely pathogenic and pathogenic) variant can be identified in approximately 9% of adult patients with CKD.^5^ Of these patients, 24% have disease-causing variants in *PKD1* and 14% in *COL4A5,* causing autosomal dominant polycystic kidney disease and X-linked Alport syndrome, respectively.^5^ The most common autosomal recessive disease genes identified were *COL4A3* in 9% and *COL4A4* in 7% of these patients with CKD.^5^

For diagnostic *a priori* probabilities, prevalence data is much needed to develop diagnostic strategies and therapy approaches for rare genetic kidney diseases. However, for nearly all ARKDs, the prevalence is either unknown, estimated to be extremely variable, dated to a pre–next-generation sequencing era, or biased toward patients with classical symptoms. This is because of many factors that influence prevalence estimation, such as the target population, selection of a representative sample, disease definition, diagnostic tests, and sample size.^6^ Based on a genotype approach, recently, disease-causing heterozygous variants in *COL4A3* and *COL4A4* have been reported in up to 1 in 106 individuals.^7^^,^^8^ Estimates of the prevalence of rare genetic kidney diseases are therefore challenging and, when available, primarily based on clinical rather than molecular genetics data.

Over the past 2 decades, significant advancements in high-throughput next-generation sequencing technologies have facilitated the generation of molecular genetics data, leading to breakthroughs in deciphering monogenic kidney diseases.^3^ Furthermore, large publicly available population-wide genetic databases such as gnomAD are now available. By determining the population frequency of disease-causing alleles in our in-house database and gnomAD, we examined 149 genes associated with ARKDs and calculated their lifetime risk.

## Methods

The methods and study design have been previously described in similar projects.^9^^,^^10^ The study was carried out in accordance with the GATHER guidelines.^11^ Sequencing of individuals in the in-house database was performed within the context of a research project approved by the ethics committee of the Klinikum rechts der Isar of the Technical University of Munich (#535/20 S). A detailed description of our in-house database can be found in the Supplementary Methods.

### Defining the Gene List

A comprehensive list of 149 genes associated with ARKDs was compiled through an extensive literature review. Genes were grouped into the following 4 ARKD subgroups: (i) glomerulopathies, (ii) tubulopathies, (iii) ciliopathies, and (iv) CAKUT. A detailed gene list can be found in Supplementary Table S1.

### Defining the Set of Pathogenic and Likely Pathogenic Variants

In the publicly available databases ClinVar (https://www.ncbi.nlm.nih.gov/clinvar/), Human Gene Mutation Database (HGMD Professional 2020.3 version, http://www.hgmd.cf.ac.uk/ac/index.php), and the Leiden Open Variation Database (LOVD, https://www.lovd.nl/), all “pathogenic” or “likely pathogenic,” and therefore disease-causing variants in 149 genes were collected if they had been reported at least once as disease-causing in one of the databases or within 1 database. In addition, our in-house exome database of the Institute of Human Genetics, as well as gnomAD v2.1.1 and SVs v2.1 (https://gnomad.broadinstitute.org/), were queried for additional frameshift, nonsense, and canonical splice-site variants not listed in any of the databases mentioned above because they can be classified as likely pathogenic. Furthermore, gnomAD was queried for copy number variations with predicted loss-of-function. For *COL4A3* and *COL4A4,* an additional search for glycine missense variants in Gly-X-Y repeats of the triple helical domain was carried out in our in-house database as well as in the gnomAD database and considered a strong criterion for pathogenicity (American College of Medical Genetics and Genomics category: PM1_strong) as recommended by the Association of Clinical Genomic Science,^12^ the Alport Variant Consortium,^13^ and as applied by others.^8^ Subsequently, all variants were reevaluated according to the guidelines of the American College of Medical Genetics and Genomics and current amendments.^12^^,^^14^, ^15^, ^16^, ^17^ Variants were excluded when downgraded to variants of uncertain significance, likely benign or benign. As recommended, the PP5 criterion was not used for variant (re)classification.

### Assessment of Allele Frequencies

Allele frequencies of disease-causing variants were assessed in 2 databases. First, gnomAD provides frequencies for different ethnic backgrounds from unrelated individuals of various disease-specific and population genetic studies. We assessed the prevalence in the European (non-Finnish) and worldwide population. The European (non-Finnish) subgroup of gnomAD was selected because it most closely aligns with our in-house database and represents the largest subgroup within gnomAD, providing a sufficient sample size for reliable assessment of allele frequencies. Furthermore, disease-causing variants might still need to be widely identified in other ethnicities.^9^ Our in-house database, "Exome Variant and Annotation Database" (EVAdb), was set up in 2010. It contains genetic data from whole exome sequencing of both healthy individuals and individuals with various genetic diseases, predominantly of Caucasian descent. As of May 2021, 23,582 individuals (e.g., 47,164 alleles) were available in the in-house database to assess minor allele frequencies. Unlike gnomAD, the genetic information in our in-house database comes from both children and adults. However, the exact ethnic composition of our in-house database was not routinely documented.^10^ The worldwide gnomAD population additionally encompasses European (Finnish), Latino/Admixed American, African/African American, Ashkenazi Jewish, East Asian, South Asian, and other populations. Second, allele frequencies were assessed in our in-house database, containing exome data from healthy and affected individuals with various genetic disorders. To avoid selection bias, cases with a kidney disease or kidney disease in their families were excluded. As of May 2021, gnomAD v2.1.1 and SVs v2.1 comprised 125,748 exomes and 15,708 genomes, respectively. Data from these 2 databases were extracted between October 2020 and December 2022.

### Estimation of the Lifetime Risks

Lifetime risk is defined as the proportion of a population that will develop the respective disease at some point in life.^9^^,^^10^ The validity and feasibility of the method have been described previously.^9^^,^^10^ In brief, the expected lifetime risk *R*_*i*_ for an ARKD caused by disease-causing variants in gene *i* was calculated from the sum of the allele frequencies *q*_*ij*_ of the n_*j*_ disease-causing variants in the respective genes under the assumption of the Hardy-Weinberg equilibrium (p^2^ + 2pq + q^2^ = 1) and mutual independence of these rare variants (Supplementary Figure S1). Biallelic combinations of variants were considered fully penetrant. The combined lifetime risk *R*_*total*_ was calculated by summation of the lifetime risks of each disease-associated gene, based on the assumption that these risks are independent and do not overlap.^9^^,^^10^

### Statistical Analysis

Statistical analysis was performed using R 4.2.0 (R Foundation for statistical computing, Vienna). Because of the low numbers, 95% CIs were calculated using the Clopper-Pearson Exact method with the GenBinomApps package.^18^ Based on the results of Groopman *et al.*,^5^ we extrapolated our estimation for autosomal recessive lifetime risk to all monogenic kidney diseases. As outlined by Groopman *et al.*,^5^ a disease-causing variant with autosomal recessive inheritance was present in 13.7% of all positive cases.

## Results

### All ARKDs

Of the total 149 genes (Supplementary Table S1), the allele frequency of 15,997 (likely) pathogenic variants was evaluated in gnomAD and our in-house database. After evaluation according to American College of Medical Genetics and Genomics and amendments, 12,912 variants comprised the final set of disease-causing variants (Supplementary Figure S1). A repository of all disease-causing variants and their allele frequency is available in the online material on figshare (Supplementary Table S2; https://doi.org/10.6084/m9.figshare.21972917). In addition, all excluded variants are available online (Supplementary Table S3; https://doi.org/10.6084/m9.figshare.21972986). The 4 disease categories included 31 genes for glomerulopathies, 16 for tubulopathies, 87 for ciliopathies, and 15 for CAKUT.

The combined estimated lifetime risk of all 149 investigated genes associated with ARKDs was 27.49 (95% confidence interval: 19.35–39.65) per 100,000 in the European, and 22.41 (17.65–28.55) per 100,000 in the worldwide gnomAD, respectively (Table 1). Supplementary Table S4 contains the individual lifetime risk for each gene as well as a supplementary description of our in-house data is provided in the Supplementary Results.

**Table 1:** Overall lifetime risk per 100,000 for 149 autosomal recessive kidney diseases and by ARKD subgroups, including 95% CIs

ARKD subgroup	European gnomAD (non-Finnish)	Worldwide gnomAD	in-house database
All 149 AR genes	27.49 (19.35–39.65)	22.41 (17.65–28.55)	10.68 (6.29–18.40)
Glomerulopathies	4.67 (3.25–6.74)	5.05 (4.01–6.39)	3.08 (1.92–4.98)
Tubulopathies	7.77 (5.86–10.39)	4.38 (3.55–5.43)	1.86 (1.21–2.90)
Ciliopathies	14.01 (9.59–20.86)	12.06 (9.43–15.46)	5.57 (3.09–10.09)
CAKUT	1.04 (0.65–1.65)	0.91 (0.65–1.28)	0.17 (0.06–0.43)

AR, autosomal recessive; ARKD, autosomal recessive kidney disease; CAKUT, congenital anomalies of the kidney and urinary tract.

### Glomerulopathies

**Figure 1:**
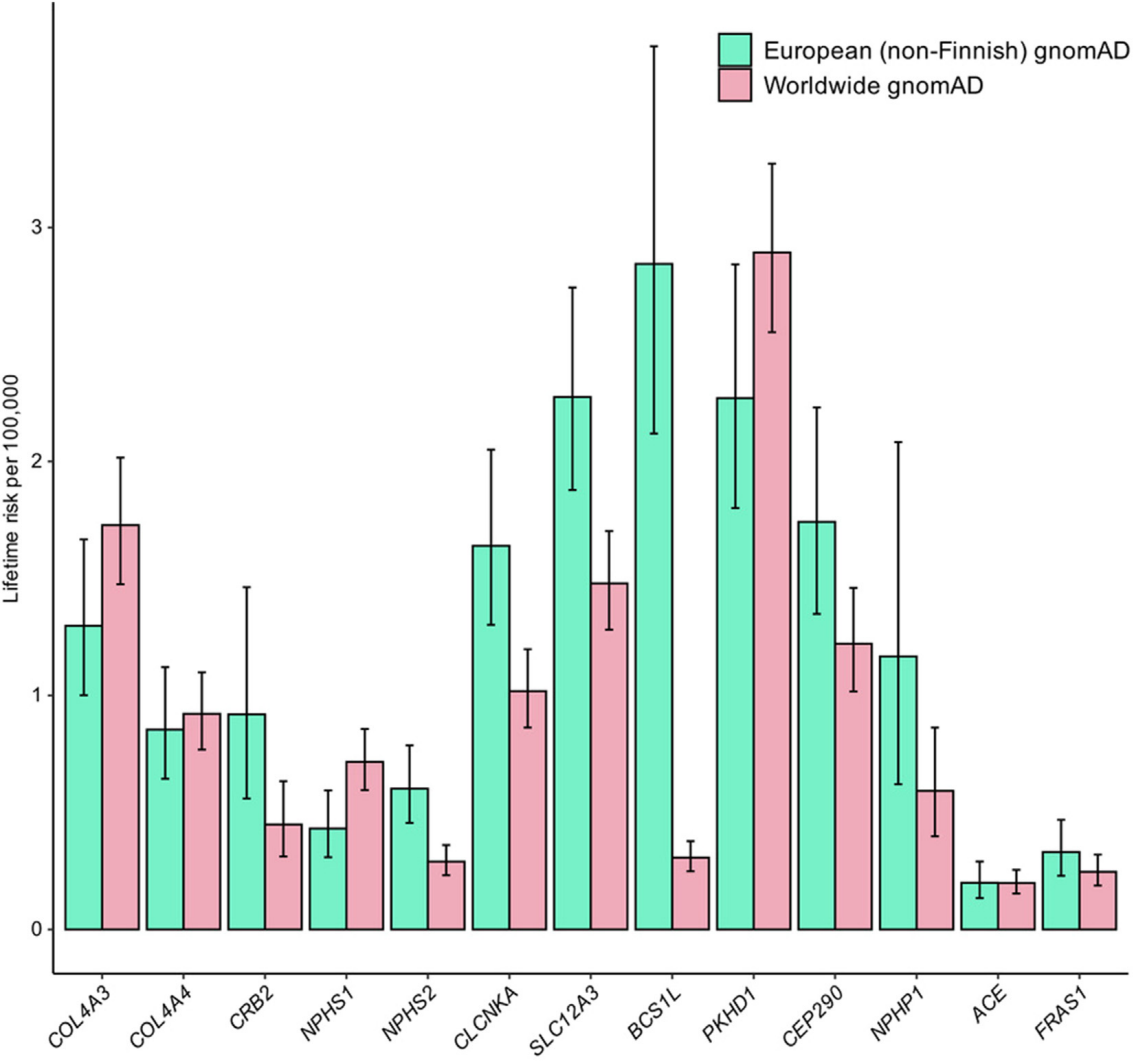
Genes with the highest calculated lifetime risk per disease subgroup of autosomal recessive kidney diseases. This figure compares the lifetime risks per 100,000 for genes linked to autosomal recessive kidney diseases, focusing on the genes with the highest lifetime risk in each disease subgroup based on the gnomAD dataset. The lifetime risk was calculated independently for the European (non-Finnish) and worldwide population. These genes' variations accounted for around 64% of the overall lifetime risk. The most pronounced lifetime risk (mean lifetime risk > 1.5 per 100,000) was observed in the 3 genes *PKHD1* (autosomal recessive polycystic kidney disease), *SLC12A3* (Gitelman syndrome), and *COL4A3* (Alport syndrome). Error bars represent 95% CIs.

The overall combined estimated lifetime risk for autosomal recessive glomerulopathies was 4.67 (3.25–6.74) per 100,000 in the European gnomAD and 5.05 (4.01–6.39) per 100,000 in the worldwide gnomAD (Supplementary Figure S2). The genes with the highest lifetime risk for monogenic glomerulopathy were *COL4A3*, followed by *COL4A4, CRB2, NPHS1,* and *NPHS2* (Table 2, Figure 1). Autosomal recessive Alport syndrome caused by disease-causing variants in *COL4A3* and *COL4A4* had a combined lifetime risk of 2.15 (1.64–2.79; European gnomAD), and 2.65 (2.24–3.12; worldwide gnomAD) per 100,000. It accounted for 9.6% (percentage of the mean lifetime risks assessed in gnomAD) of the overall lifetime risk and for 49.4% of glomerulopathies.

**Table 2:** Lifetime risk for the most frequent autosomal recessive kidney diseases by subgroup

Gene	Number of disease-causing variants in-house database	Number of disease-causing alleles in-house database	Number of disease-causing variants in gnomAD dataset	Number of disease-causing alleles in European (non-Finnish) gnomAD	Number of disease-causing alleles in worldwide gnomAD	Lifetime risk European (non-Finnish) gnomAD per 100,000 (95% CI)	Lifetime risk worldwide gnomAD per 100,000 (95% CI)	Lifetime risk in-house per 100,000 (95% CI)
Glomerulopathies								
*COL4A3*	63	158	275	316	833	1.30 (1.00–1.67)	1.73 (1.48–2.02)	1.11 (0.77–1.59)
*COL4A4*	50	114	247	268	638	0.85 (0.64–1.12)	0.92 (0.77–1.10)	0.58 (0.37–0.88)
*CRB2*	15	83	67	91	165	0.92 (0.56–1.46)	0.45 (0.31–0.63)	0.36 (0.21–0.58)
*NPHS1*	20	57	90	13	619	0.43 (0.31–0.59)	0.72 (0.60–0.86)	0.15 (0.08–0.26)
*NPHS2*	23	115	53	276	417	0.60 (0.46–0.79)	0.29 (0.23–0.36)	0.58 (0.37–0.87)
Tubulopathies								
*SLC12A3*	52	184	150	567	1004	2.28 (1.88–2.74)	1.48 (1.23–1.78)	1.48 (1.05–2.05)
*CLCNKA*	5	7	67	397	758	1.64 (1.30–2.05)	1.02 (0.86–1.20)	0.00 (0.00–0.01)
*BCS1L*	22	55	61	247	469	2.84 (2.12–3.77)	0.31 (0.25–0.38)	0.13 (0.07–0.24)
Ciliopathies								
*PKHD1*	45	169	221	393	1310	2.27 (1.80–2.84)	2.89 (2.55–3.27)	1.28 (0.84–1.91)
*CEP290*	56	111	202	324	625	1.74 (1.35–2.23)	1.22 (1.02–1.46)	0.59 (0.38–0.90)
*NPHP1*	9	13	54	58	139	1.17 (0.62–2.08)	0.59 (0.40–0.86)	0.81 (0.54–1.20)
CAKUT								
*ACE*	16	26	96	139	320	0.20 (0.13–0.29)	0.20 (0.15–0.25)	0.03 (0.01–0.08)
*FRAS1*	27	41	171	163	290	0.33 (0.23–0.47)	0.25 (0.19–0.32)	0.06 (0.03–0.13)

CAKUT, congenital anomalies of the kidney and urinary tract.

### Tubulopathies

The combined lifetime risk for autosomal recessive tubulopathies was 7.77 (5.86–10.39) per 100,000 in the European gnomAD, and 4.38 (3.55–5.42) per 100,000 in worldwide gnomAD (Supplementary Figure S3). This difference was primarily because of a single disease-causing variant in *BCS1L* NM_001079866.2: c.232A>G; p.(Ser78Gly) associated with GRACILE syndrome.^19^, ^20^, ^21^ This variant has a higher frequency in the European gnomAD (minor allele frequency European = 0.004062, minor allele frequency worldwide = 0.000474).

The genes with the highest lifetime risk for tubulopathies were *SLC12A3, CLCNKA,* and *BCS1L* (Table 2, Figure 1). Gitelman and Bartter syndromes represent the 2 most prevalent tubulopathies. Disease-causing variants in the *SLC12A3* associated with Gitelman syndrome had the highest contribution of a single gene to the overall calculated lifetime risk of ARKDs in the European gnomAD with 2.28 (1.88–2.74; European gnomAD), and based on the whole gnomAD dataset the second largest contribution with 1.48 (1.23–1.78; worldwide gnomAD) per 100,000, respectively. The combined lifetime risk of 10 genes associated with Bartter syndrome was 2.41 (1.78–3.29; European gnomAD), and 2.50 (1.97–3.17, worldwide gnomAD) per 100,000.

### Ciliopathies

The autosomal recessive ciliopathy subgroup comprised the largest ARKD subgroup and accounted for the highest overall lifetime risk of the ARKD subgroups (Table 1). Based on the allele frequencies of 87 genes, the combined lifetime risk was 14.01 (9.59–20.86; European gnomAD), and 12.06 (9.43–15.46; worldwide gnomAD) per 100,000 (Supplementary Figure S4). The genes with the highest lifetime risk were *PKHD1*, *CEP290*, and *NPHP1* (Table 2, Figure 1). Based on the whole gnomAD dataset, *PKHD1* had the highest single gene contribution to the overall lifetime risk of ARKD with 2.89 (2.55–3.27; worldwide gnomAD); in the European gnomAD it was 2.27 (1.80–2.84; European gnomAD) per 100,000.

### CAKUT

The combined lifetime risk of CAKUT was 1.04 (0.65–1.65), and 0.91 (0.65–1.28) per 100,000 in the European and worldwide gnomAD, respectively (Supplementary Figure S5). Autosomal recessive CAKUT had the lowest mean contribution to the overall lifetime risk as calculated based on the gnomAD database, with 0.98 per 100,000 (3.91%). In this ARKD subgroup, *ACE*-and *FRAS1*-associated CAKUT had the highest mean lifetime risk (Table 2, Figure 1).

### Extrapolation to All Monogenic Kidney Diseases

**Figure 2:**
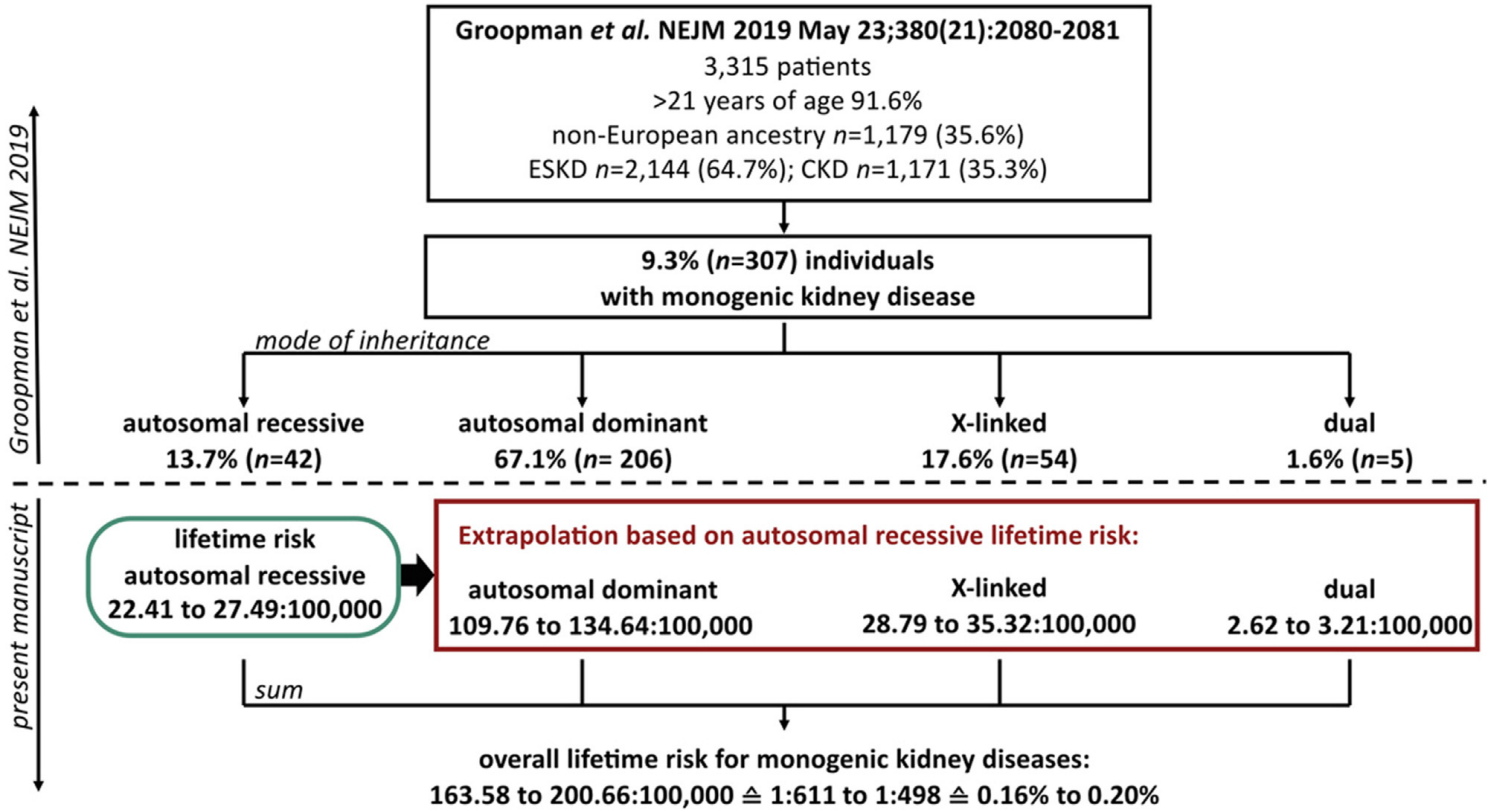
Extrapolation of autosomal recessive lifetime risk to all monogenic kidney disease. To calculate the overall lifetime risk for a monogenic kidney disease, we extrapolated our data based on the distribution of inheritance types as observed by Groopman *et al.*^5^ Summation of all types of inheritances yielded a total lifetime risk of 1 in 611 to 1 in 498 adult individuals. This would equal 0.16% to 0.20% of the adult population. CKD, chronic kidney disease; ESKD, end-stage kidney disease.

Based on the results of Groopman *et al.*,^5^ we extrapolated the lifetime risk for autosomal recessive genes to all monogenic kidney diseases (Figure 2). Of individuals in this study, 91.6% were aged > 21 years, 64.7% had end-stage kidney disease, and 35.6% were of non-European ancestry.^5^ Autosomal recessive inheritance was present in 13.7% of all genetically solved cases.^5^ Extrapolation based on the lowest and highest ARKD lifetime risk yielded a lifetime risk of 110 (lower extrapolation) to 134 (upper extrapolation) per 100,000 individuals for autosomal dominant inheritance, 29 to 35 per 100,000 individuals for X-linked inheritance, and of 3 per 100,000 for dual causes of a monogenic kidney disease. This sums up to an overall lifetime risk for a monogenic kidney disease of 164 to 201 per 100,000, representing 1 in 611 (0.16%) to 1 in 498 (0.20%). Based on an observed CKD prevalence of 12% in Europe,^4^ genetic kidney disease may affect between 1.37% to 1.68% of adult patients with CKD, equivalent to 1 in 73 and 1 in 60 individuals, assuming their mortality rate matches that of the general population.

## Discussion

This study provides frequencies of ARKDs in 2 independent databases using a population-based genotype approach primarily focusing on non-Finnish European populations. These findings will aid in evaluating diagnostic *a priori* probabilities, thereby complementing existing epidemiological data. The overall combined lifetime risk for ARKD was estimated at 27.49 per 100,000 based on the European gnomAD dataset and 22.41 per 100,000 based on the worldwide gnomAD dataset. This translates to an individual lifetime risk of 1 in 3638 to 1 in 4462 individuals.

The highest lifetime risk (> 1.5 per 100,000) was estimated for nephropathies caused by variants in *PKHD1, SLC12A3*, and *COL4A3* associated with autosomal recessive polycystic kidney disease, Gitelman syndrome, and Alport syndrome, respectively. Disease-causing variants in these 3 genes accounted for up to 24% of the overall lifetime risk. Among the databases analyzed, European (non-Finnish) gnomAD represents the largest population subgroup and, as such, offers the most precise population data. Therefore, our results should be interpreted with the understanding that the estimation for the non-Finnish European population is the most precise. For most genes, the lifetime risk calculated from allele frequencies was systemically higher in gnomAD compared to our in-house database. This discrepancy can be attributed to the larger size of the gnomAD dataset, which includes numerous rare loss-of-function variants absent in our in-house database. In addition, we rigorously excluded cases associated with kidney disease from our in-house dataset. However, the precise composition of both gnomAD and our in-house database is unknown, and the observed differences may reflect the diversity within the European population.

One prominent example of an underestimated prevalence is the contribution of autosomal recessive Alport syndrome variants that accounted for 10% of the overall lifetime risk. The calculated lifetime risk is similar to recently published data based on the gnomAD dataset, where a predicted disease-causing variant in *COL4A3* and *COL4A4* occurred in 1 of 106 individuals,^8^ equal to a lifetime risk of 2.2 per 100,000. Considering that a relevant amount of heterozygous disease-causing variants in *COL4A3* and *COL4A4* also cause thin basement membrane nephropathy (now mostly described as autosomal dominant Alport syndrome) with a lower frequency of classic symptoms and a lower probability of causing end-stage kidney disease, the actual lifetime risk would be even higher.^7^ Therefore, the provision of molecular genetics data is valuable because the prevalence of milder forms of Alport syndrome is unknown and underdiagnosed.^7^^,^^22^

Although we can estimate the lifetime risk for a monogenic kidney disease, we are unable to report the likelihood of moderate to severe CKD because large genotype-phenotype population-wide studies are not available for extrapolation. For example, for Gitelman syndrome, a common genetic tubulopathy caused by disease-causing variants in *SLC12A3*, the estimated prevalence in Asia is 1 in 40,000,^23^ comparable to our range of 1 in 43,860 (gnomAD European). Treatment consists of electrolyte supplementation to prevent rare complications such as life-threatening arrhythmias because of hypokalemia. Gitelman syndrome has been previously termed benign tubulopathy; however, current reports describe renal dysfunction in up to 20% of adult patients.^24^

Ciliopathies, the subgroup with the most genes examined in the current study, were responsible for half of the overall lifetime risk. In this subgroup, *PKHD1*-associated autosomal recessive polycystic kidney disease had the most substantial contribution of a single diagnosis to the overall lifetime risk. The incidence of autosomal recessive polycystic kidney disease is regionally different and has been described in Europe with up to 1:20,000.^25^^,^^26^ However, initial incidence estimations were solely based on clinical diagnosis because molecular genetic analyses were not available.^25^ Today, it is known that multiple genes cause ciliopathies, which can mimic the phenotype of polycystic kidney disease (“phenocopy”). We found a lower lifetime risk of 1 in 34,602 to 1 in 44,052 for *PKHD1* alone; however*,* when combining all ciliopathy genes, lifetime risk was higher between 1 in 7138 to 1 in 8292. Therefore, phenocopies and differences in disease definition might hamper comparability to previous, especially older estimations.^25^ Determining the frequency of rare monogenic diseases once required examining large populations, a highly resource-intensive process. However, the presented data highlights the effectiveness of population-based approaches in accurately estimating their prevalence.

CAKUT comprises a diverse spectrum of renal and urinary tract malformations. The contribution of monogenic variants to the disease spectrum is small, because monogenic forms in children have been estimated to be responsible for only approximately 16% of cases.^27^ Furthermore, most disease-associated genes have an autosomal dominant inheritance or pathogenicity caused by copy number variations.^27^ CAKUT has been observed in 3 to 6 per 1000 pregnancies.^28^ Consequently, the autosomal recessive CAKUT subgroup, with a monogenic ARKD lifetime risk of 0.0091 to 0.0104 per 1000 pregnancies, contributes minimally to the overall CAKUT disease spectrum. In addition, the CAKUT disease subgroup made the smallest contribution to the overall monogenic ARKD lifetime risk, accounting for just 4%. For this subgroup, alternative causes, such as polygenic and autosomal dominant inheritance, are likely of greater significance.

One limitation of our method to calculate the lifetime risk is that it can only be used for autosomal recessive diseases. Therefore, we extrapolated the calculation of autosomal recessive lifetime risk to all monogenic kidney diseases, based on the distribution of inheritance types observed by Groopman *et al.*^5^ Summation of all types of inheritances was equal to a total lifetime risk of 1 in 611 to 1 in 498 adult individuals (Figure 2). This would be equal to the presence of a monogenic kidney disease in adult patients with CKD of 1 in 73 to 1 in 60. We are aware that this extrapolation can only be a rough estimation of the overall lifetime risk of monogenic kidney disease. Primarily, because the sequencing data from Groopman *et al.*^5^ was not assessed in a representative cohort and individuals were included based on a clinical phenotype. Moreover, it had an accumulation of individuals with end-stage kidney disease^5^; therefore, mild to moderately affected individuals might be underrepresented in the cohort. Most cases, that is, 92% were aged > 21 years, and with 14% autosomal recessive inheritance, had the smallest contribution to the overall prevalence of a genetic disease cause.^5^ Furthermore, population-based observational studies or sequencing studies in adult patients with CKD might not reflect the actual disease occurrence because some monogenic kidney diseases are associated with high mortality at younger ages or lethal during embryonic development. Therefore, the assumption of comparable mortality to the general population is imprecise and further leads to an underestimation of the real lifetime risk.

Finally, several limitations must be considered when interpreting our results. Underestimation of the lifetime risk may have occurred because of excluding variants not identified by exome sequencing, such as intronic splice variants. To date, there is no clear evidence of disease-causing intronic variants identified by genome sequencing for genetic kidney disease. Oligogenic or polygenic inheritance and risk scores for genetic kidney diseases might become of relevance with further research.^29^ Furthermore, gnomAD v2.1.1 and SVs v2.1 as well as our in-house database contain primarily whole exome instead of whole genome sequencing data. At the time we began our assessment, only gnomAD v2.1 was available. Unlike gnomAD v2.1, the more recent gnomAD v4.1 also includes individuals from the UK Biobank, thereby incorporating a broader spectrum of health conditions, including those with underlying diseases. Our estimation relies on the assumption that individuals with diseases, particularly kidney disease, are excluded. However, as genomic data continues to expand, it is essential to validate our findings using larger, more diverse as well as lager non-European databases. Our approach therefore might introduce an ascertainment bias toward healthy individuals. Nevertheless, this could eventually contribute to genetic lifetime risk. Moreover, genetic risk for kidney disease comes from frequent risk alleles in genes such as *APOL1*^30^ or *de novo* variants in individuals without a positive family history. In addition, in some populations, lifetime risk could be driven by founder variants such as in *NPHS1* and *NPHS2*.^31^ We focused on the 4 disease categories, glomerulopathies, tubulopathies, ciliopathies, and CAKUT comprising 149 genes. To maintain an overview of the many reported genes, we chose to report the lifetime risk of 4 clinical disease entities. However, we would like to point out that this division is artificial and that with further knowledge, it might become important to focus on the lifetime risk of each single gene; especially in the era of precision medicine when gene-specific therapies, such as antisense oligonucleotide therapy, become available. The discovery of new disease-causing variants in the examined genes, identification of new genes, as well as additional disease categories associated with genetic kidney diseases could add additional lifetime risk.^9^ It has to be noted that this study naturally contributes little to the epidemiological knowledge of monogenic kidney diseases with predominantly autosomal dominant inheritance, such as CAKUT.^32^ Conversely, the lifetime risk in this study could be overestimated if benign variants are misinterpreted as disease-causing, or in the cases of (likely) pathogenic variants with reduced penetrance, which, though occasionally implicated in autosomal recessive disorders, do not consistently lead to disease.^10^^,^^33^ We tried to minimize sampling error from imperfect random samples by conducting the calculations in 2 independent databases (gnomAD and in-house).

## Conclusion

In conclusion, we provide a general population-genetic estimation for the lifetime risk of ARKDs across various populations, with a primary focus on European populations. Our findings suggest a substantial cumulative prevalence of ARKDs, highlighting the importance of monogenic causes of CKD. The data will be essential for clinical, genetic, and therapeutic considerations and resource allocation in therapy development, public health management, and biomedical research in genetic kidney diseases.

## Disclosure

MCB reports travel support from Lilly Germany and consulting fees from Boehringer Ingelheim and Samsung Bioepis unrelated to the project. All the other authors declared no competing interests.

## Supplementary Material

Supplementary File (PDF) **Supplementary Methods. Supplementary References. Figure S1.** Flowchart of the analysis. **Figure S2.** Calculated lifetime risk of genes associated with autosomal recessive glomerulopathies. **Figure S3.** Calculated lifetime risk of genes associated with autosomal recessive tubulopathies. **Figure S4.** Calculated lifetime risk of genes associated with autosomal recessive ciliopathies. **Figure S5.** Calculated lifetime risk of genes associated with autosomal recessive CAKUT. **Table S1.** List of 149 genes associated with an autosomal recessive kidney disease. **Table S2.** Detailed list of included variants and their allele frequencies. https://doi.org/10.6084/m9.figshare.21972917. **Table S3.** Detailed list of excluded variants. https://doi.org/10.6084/m9.figshare.21972986. **Table S4.** List of the lifetime risk of all 149 genes associated with an autosomal recessive kidney disease. **GATHER Checklist.**

## Footnotes

Supplementary File (PDF)

**Supplementary Methods.**

**Supplementary References.**
**Figure S1.** Flowchart of the analysis. **Figure S2.** Calculated lifetime risk of genes associated with autosomal recessive glomerulopathies. **Figure S3.** Calculated lifetime risk of genes associated with autosomal recessive tubulopathies. **Figure S4.** Calculated lifetime risk of genes associated with autosomal recessive ciliopathies. **Figure S5.** Calculated lifetime risk of genes associated with autosomal recessive CAKUT. **Table S1.** List of 149 genes associated with an autosomal recessive kidney disease. **Table S2.** Detailed list of included variants and their allele frequencies. https://doi.org/10.6084/m9.figshare.21972917. **Table S3.** Detailed list of excluded variants. https://doi.org/10.6084/m9.figshare.21972986. **Table S4.** List of the lifetime risk of all 149 genes associated with an autosomal recessive kidney disease.
**GATHER Checklist.**
