# Supplementary material for: Estimating Lifetime Risk of Autosomal Recessive Kidney Diseases Using Population-Based Genotypic Data
Source: Kidney Int Rep. 2025 Apr 21;10(7):2384–93. doi: 10.1016/j.ekir.2025.04.036 (PMC12266173; doi:10.1016/j.ekir.2025.04.036)
Supplement: Supplementary File (PDF) — Supplementary Methods. Supplementary References. Figure S1. Flowchart of the analysis. Figure S2. Calculated lifetime risk of genes associated with autosomal recessive glomerulopathies. Figure S3. Calculated lifetime risk of genes associated with autosomal recessive tubulopathies. Figure S4. Calculated lifetime risk of genes associated with autosomal recessive ciliopathies. Figure S5. Calculated lifetime risk of genes associated with autosomal recessive CAKUT. Table S1. List of 149 genes associated with an autosomal recessive kidney disease. Table S2. Detailed list of included variants and their allele frequencies. https://doi.org/10.6084/m9.figshare.21972917. Table S3. Detailed list of excluded variants. https://doi.org/10.6084/m9.figshare.21972986. Table S4. List of the lifetime risk of all 149 genes associated with an autosomal recessive kidney disease. GATHER Checklist. [file mmc1.pdf]

Supplementary Material

Estimating Lifetime Risk of Autosomal Recessive Kidney Diseases Using Population-Based Genotypic Data

Matthias C. Braunisch[1,#], Clara M. Großewinkelmann[2], Martin Menke[2], Nora Hannane[1], Riccardo Berutti[2], Jasmina Ćomić[1,2], Roman Günthner[1], Lutz Renders[1], Christoph Schmaderer[1], Uwe Heemann[1], Korbinian M. Riedhammer[1,2,3], Matias Wagner[2,4,*], and Julia Hoefele[2,5,#,*]

[1] Department of Nephrology, Klinikum rechts der Isar, Technical University of Munich, TUM School of Medicine and Health, Ismaninger Straße 22, Munich, Germany

[2] Institute of Human Genetics, Klinikum rechts der Isar, Technical University of Munich, TUM School of Medicine and Health, Trogerstraße 32, Munich, Germany

[3] Division of Nephrology, Boston Children's Hospital, Harvard Medical School, Boston, 02115, United States of America

[4] Institute of Neurogenomics, Helmholtz Zentrum München, Ingolstädter Landstraße 1, Neuherberg, Germany

[5] Institute of Human Genetics, University Hospital, Ludwig-Maximilians University, Munich, Germany

* these authors contributed equally to the work

Running title: Lifetime risk of kidney diseases

[#] Correspondence to:

PD Dr. med. Matthias Christoph Braunisch, Department of Nephrology, Klinikum rechts der Isar, Technical University of Munich, TUM School of Medicine and Health, Ismaninger Str. 22, 81675 Munich, Germany. Email: matthias.braunisch@mri.tum.de, Phone + 49 (0) 89 4140-2231; Fax: + 49 (0) 89 4140-7734

and

Prof. Dr. med. Julia Hoefele, Institute of Human Genetics, University Hospital, Ludwig-Maximilians University, Goethestr. 29, 80336 Munich, Germany. Email: julia.hoefele@med.uni-muenchen.de, Phone: + 49 (0) 89 4400-53683, Fax: + 49 (0) 89 4400-54468.

Supplement

Supplementary Methods

Supplementary Table 1. List of 149 genes associated with an autosomal recessive kidney disease.

Supplementary Table 2. Detailed list of included variants and their allele frequencies. https://doi.org/10.6084/m9.figshare.21972917

Supplementary Table 3. Detailed list of excluded variants. https://doi.org/10.6084/m9.figshare.21972986

Supplementary Table 4. List of the lifetime risk of all 149 genes associated with an autosomal recessive kidney disease.

Supplementary Figure 1. Flowchart of the analysis.

Supplementary Figure 2. Calculated lifetime risk of genes associated with autosomal recessive glomerulopathies.

Supplementary Figure 3. Calculated lifetime risk of genes associated with autosomal recessive tubulopathies.

Supplementary Figure 4. Calculated lifetime risk of genes associated with autosomal recessive ciliopathies.

Supplementary Figure 5. Calculated lifetime risk of genes associated with autosomal recessive CAKUT.

GATHER Checklist

Supplementary References

Supplement

Supplementary Methods

Whole exome sequencing data in our in-house database was obtained with the Sure Select Human All Exon V5 (50 Mb) Kit (Agilent Technologies, Inc., Santa Clara, CA, United States of America) and a HiSeq2500 (Illumina, Inc., San Diego, CA, United States of America) or with Sure Select Human All Exon V6 (60 Mb) Kit (Agilent Technologies, Inc., Santa Clara, CA, United States of America) and a HiSeq4000 (Illumina). Alignment of reads was done according to the Genome Reference Consortium Human Build 37 (UCSC Genome Browser build hg19) using Burrows-Wheeler Aligner (v.0.7.5a). SAMtools (version 0.1.19) was employed for detection of single-nucleotide variants (SNVs) and small insertions and deletions (indels) [S1, S2]. More than 95% of target sequences are at least covered >20 times. The reads are aligned to the Human Genome Assembly GRCh37 (hg19). For exomes and genomes, SNVs (single nucleoid variants) and indels (insertions/deletions) that deviate from the reference genome are determined using the Genome Analysis Toolkit 4 (GATK 4.2.3.0; https://github.com/broadinstitute/gatk). The detailed algorithms correspond to the recommendations of "GATK Best Practices" of the Broad Institute according to the publications [S3, S4]. The GATK tools HaplotypeCaller and GenotypeGVCFs are used. The resulting variant files (VCFs) are further processed with the GATK Variant Quality Recalibration Tools (VariantRecalibrator and ApplyVQSR). The data sets on which these tools are based are in the GATK Resource Bundle (https://gatk.broadinstitute.org/hc/en-us/articles/360035890811 - Resource-bundle). Mitochondrial variants are called with GATK4 Haplotypecaller and GenotypeGVCFs. In addition, exonic variants are also called with samtools, the resulting VCFs are post-processed and filtered with user-defined scripts (part of the pipeline, see Github). Indels up to 20 kbp are analyzed with Pindel Caller [S5]. For CNVs (Copy Number Variation, exon-wise) the tool ExomeDepth [S6] is used. For autosomes, a reference dataset with 50 samples is created; for the X chromosome, two additional reference datasets are created separately for male and female samples.

In HGMD, variants classified as DM (disease-causing mutation) and DM? (questionable disease-causing mutation) were included for reevaluation, while those classified as DP (disease-associated polymorphism) were not considered.

Supplementary results of the in-house database

As of May 2021, our inhouse database comprised 23,582 individuals. The combined estimated lifetime risk of all 149 investigated genes associated with autosomal recessive kidney diseases was 10.68 (95% CI 6.29-18.40) in our in-house database.

The overall combined estimated lifetime risk for autosomal recessive glomerulopathies was 3.08 (1.92-4.98) per 100,000 in our in-house database. Autosomal recessive Alport syndrome caused by disease-causing variants in *COL4A3* and *COL4A4* had a combined in-house lifetime risk of 1.69 (1.14-2.46).

The combined lifetime risk for autosomal recessive tubulopathies was 1.86 (1.21-2.90) in-house. The *CLCNKA* gene contained only a few disease-causing alleles in our in-house database (MAF = 0.000156), whereas these alleles were notably more frequent in the gnomAD dataset (MAF European = 0.003578, MAF worldwide = 0.002870).

The autosomal recessive ciliopathy subgroup represented the highest overall lifetime risk among the autosomal recessive kidney disease subgroups (**Table 1**), which is also reflected in our in-house database with a combined lifetime risk of 5.57 (3.09-10.09). *PKHD1* had an in-house lifetime risk of 1.28 (0.84-1.91).

The combined lifetime risk of CAKUT was lower in the in-house database with 0.17 (0.06-0.43) per 100,000 compared to gnomAD.

Supplementary Table 1. List of 149 genes associated with autosomal recessive kidney disease.

Gene#	Disease group	Disease subgroup	OMIM	Gene	Protein
1	Glomerulopathies	FSGS/SNRS	* 601925	*ARHGDIA*	RHO GDP-DISSOCIATION INHIBITOR ALPHA
2	Glomerulopathies	AS/TBMN	* 120070	*COL4A3*	COLLAGEN, TYPE IV, ALPHA-3
3	Glomerulopathies	AS/TBMN	* 120131	*COL4A4*	COLLAGEN, TYPE IV, ALPHA-4
4	Glomerulopathies	FSGS/SNRS	* 609825	*COQ2*	COENZYME Q2, POLYPRENYLTRANSFERASE
5	Glomerulopathies	FSGS/SNRS	* 614647	*COQ6*	COENZYME Q6, MONOOXYGENASE
6	Glomerulopathies	FSGS/SNRS	* 615567	*COQ8B*	COENZYME Q8B
7	Glomerulopathies	FSGS/SNRS	* 609720	*CRB2*	CRUMBS CELL POLARITY COMPLEX COMPONENT 2
8	Glomerulopathies	FSGS/SNRS	* 606627	*DAAM2*	DISHEVELLED-ASSOCIATED ACTIVATOR OF MORPHOGENESIS 2
9	Glomerulopathies	FSGS/SNRS	* 601440	*DGKE*	DIACYLGLYCEROL KINASE, EPSILON, 64-KD
10	Glomerulopathies	FSGS/SNRS	* 602334	*EMP2*	EPITHELIAL MEMBRANE PROTEIN 2
11	Glomerulopathies	FSGS/SNRS	* 605025	*ITGA3*	INTEGRIN, ALPHA-3
12	Glomerulopathies	FSGS/SNRS	* 607704	*KANK1*	KN MOTIF- AND ANKYRIN REPEAT DOMAIN-CONTAINING PROTEIN
13	Glomerulopathies	FSGS/SNRS	* 614610	*KANK2*	KN MOTIF- AND ANKYRIN REPEAT DOMAIN-CONTAINING PROTEIN
14	Glomerulopathies	FSGS/SNRS	* 614612	*KANK4*	KN MOTIF- AND ANKYRIN REPEAT DOMAIN-CONTAINING PROTEIN 4
15	Glomerulopathies	FSGS/SNRS	* 150325	*LAMB2*	LAMININ, BETA-2
16	Glomerulopathies	FSGS/SNRS	* 601479	*MYO1E*	MYOSIN IE
17	Glomerulopathies	FSGS/SNRS	* 602716	*NPHS1*	NEPHRIN
18	Glomerulopathies	FSGS/SNRS	* 604766	*NPHS2*	PODOCIN
19	Glomerulopathies	FSGS/SNRS	* 607617	*NUP107*	NUCLEOPORIN, 107-KD
20	Glomerulopathies	FSGS/SNRS	* 607613	*NUP133*	NUCLEOPORIN, 133-KD
21	Glomerulopathies	FSGS/SNRS	* 607614	*NUP160*	NUCLEOPORIN, 160-KD
22	Glomerulopathies	FSGS/SNRS	* 614352	*NUP205*	NUCLEOPORIN, 205-KD
23	Glomerulopathies	FSGS/SNRS	* 170285	*NUP85*	NUCLEOPORIN, 85-KD
24	Glomerulopathies	FSGS/SNRS	* 614351	*NUP93*	NUCLEOPORIN, 93-KD
25	Glomerulopathies	FSGS/SNRS	* 610564	*PDSS2*	PRENYL DIPHOSPHATE SYNTHASE, SUBUNIT 2
26	Glomerulopathies	FSGS/SNRS	* 608414	*PLCE1*	PHOSPHOLIPASE C, EPSILON-1
27	Glomerulopathies	FSGS/SNRS	* 600579	*PTPRO*	PROTEIN-TYROSINE PHOSPHATASE, RECEPTOR-TYPE, O
28	Glomerulopathies	FSGS/SNRS	* 603729	*SGPL1*	SPHINGOSINE-1-PHOSPHATE LYASE 1
29	Glomerulopathies	FSGS/SNRS	* 606622	*SMARCAL1*	SWI/SNF-RELATED, MATRIX-ASSOCIATED, ACTIN-DEPENDENT REGULATOR OF CHROMATIN, SUBFAMILY A-LIKE PROTEIN 1
30	Glomerulopathies	FSGS/SNRS	* 606125	*TRIM8*	TRIPARTITE MOTIF-CONTAINING PROTEIN 8
31	Glomerulopathies	FSGS/SNRS	* 616144	*WDR73*	WD REPEAT-CONTAINING PROTEIN 73
32	Tubulopathies	Bartter syndrome	* 606412	*BSND*	BSND GENE
33	Tubulopathies	Bartter syndrome	* 602024	*CLCNKA*	CHLORIDE CHANNEL, KIDNEY, A

34	Tubulopathies	Bartter syndrome	* 602023	*CLCNKB*	CHLORIDE CHANNEL, KIDNEY, B
35	Tubulopathies	Bartter syndrome	* 603959	*CLDN16*	CLAUDIN 16
36	Tubulopathies	Bartter syndrome	* 610036	*CLDN19*	CLAUDIN 19
37	Tubulopathies	Bartter syndrome	* 607803	*CNNM2*	CYCLIN M2
38	Tubulopathies	Bartter syndrome	* 131530	*EGF*	EPIDERMAL GROWTH FACTOR
39	Tubulopathies	Bartter syndrome	* 600359	*KCNJ1*	POTASSIUM CHANNEL, INWARDLY RECTIFYING, SUBFAMILY J, MEMBER 1
40	Tubulopathies	Bartter syndrome	* 600839	*SLC12A1*	SOLUTE CARRIER FAMILY 12 (SODIUM/POTASSIUM/CHLORIDE TRANSPORTER), MEMBER
41	Tubulopathies	Bartter syndrome	* 607009	*TRPM6*	TRANSIENT RECEPTOR POTENTIAL CATION CHANNEL, SUBFAMILY M, MEMBER 6
42	Tubulopathies	Gitelman syndrome	* 600968	*SLC12A3*	SOLUTE CARRIER FAMILY 12 (SODIUM/CHLORIDE TRANSPORTER), MEMBER 3
43	Tubulopathies	Renal tubular acidosis	* 605239	*ATP6V0A4*	ATPase, H+ TRANSPORTING, LYSOSOMAL, V0 SUBUNIT A, ISOFORM 4
44	Tubulopathies	Renal tubular acidosis	* 192132	*ATP6V1B1*	ATPase, H+ TRANSPORTING, LYSOSOMAL, 56/58-KD, V1 SUBUNIT B, ISOFORM 1
45	Tubulopathies	Renal tubular acidosis	* 603647	*BCS1L*	BCS1 HOMOLOG, UBIQUINOL-CYTOCHROME C REDUCTASE COMPLEX CHAPERONE
46	Tubulopathies	Renal tubular acidosis	* 611492	*CA2*	CARBONIC ANHYDRASE II
47	Tubulopathies	Renal tubular acidosis	* 603345	*SLC4A4*	SOLUTE CARRIER FAMILY 4 (SODIUM BICARBONATE COTRANSPORTER), MEMBER 4
48	Ciliopathies	Polycystic kidney disease	* 617570	*DZIP1L*	DAZ-INTERACTING ZINC FINGER PROTEIN 1-LIKE
49	Ciliopathies	Polycystic kidney disease	* 606702	*PKHD1*	PKHD1 CILIARY IPT DOMAIN-CONTAINING FIBROCYSTIN/POLYDUCTIN
50	Ciliopathies	Nephronophthisis	* 608894	*AHI1*	ABELSON HELPER INTEGRATION SITE 1
51	Ciliopathies	Nephronophthisis	* 606844	*ALMS1*	ALMS1 CENTROSOME AND BASAL BODY ASSOCIATED PROTEIN
52	Ciliopathies	Nephronophthisis	* 615370	*ANKS6*	ANKYRIN REPEAT AND STERILE ALPHA MOTIF DOMAINS-CONTAINING PROTEIN 6
53	Ciliopathies	Nephronophthisis	* 608922	*ARL13B*	ADP-RIBOSYLATION FACTOR-LIKE 13B
54	Ciliopathies	Nephronophthisis	* 604695	*ARL3*	ADP-RIBOSYLATION FACTOR-LIKE 3
55	Ciliopathies	Nephronophthisis	* 608845	*ARL6*	ADP-RIBOSYLATION FACTOR-LIKE 6
56	Ciliopathies	Nephronophthisis	* 617612	*ARMC9*	ARMADILLO REPEAT-CONTAINING PROTEIN 9
57	Ciliopathies	Nephronophthisis	* 614144	*B9D1*	B9 DOMAIN-CONTAINING PROTEIN 1
58	Ciliopathies	Nephronophthisis	* 611951	*B9D2*	B9 DOMAIN-CONTAINING PROTEIN 2
59	Ciliopathies	Nephronophthisis	* 613605	*BBIP1*	BBS PROTEIN COMPLEX-INTERACTING PROTEIN 1
60	Ciliopathies	Nephronophthisis	* 209901	*BBS1*	BBS1 GENE
61	Ciliopathies	Nephronophthisis	* 610148	*BBS10*	BBS10 GENE
62	Ciliopathies	Nephronophthisis	* 610683	*BBS12*	BBS12 GENE
63	Ciliopathies	Nephronophthisis	* 606151	*BBS2*	BBS2 GEN
64	Ciliopathies	Nephronophthisis	* 600374	*BBS4*	BBS4 GEN
65	Ciliopathies	Nephronophthisis	* 603650	*BBS5*	BBS5 GEN
66	Ciliopathies	Nephronophthisis	* 607590	*BBS7*	BBS7 GEN
67	Ciliopathies	Nephronophthisis	* 607968	*BBS9*	BBS9 GEN
68	Ciliopathies	Nephronophthisis	* 615944	*C2CD3*	C2 CALCIUM-DEPENDENT DOMAIN-CONTAINING PROTEIN 3
69	Ciliopathies	Nephronophthisis	* 614477	*C8orf37*	CHROMOSOME 8 OPEN READING FRAME 37
70	Ciliopathies	Nephronophthisis	* 612013	*CC2D2A*	COILED-COIL AND C2 DOMAINS-CONTAINING PROTEIN 2A

71	Ciliopathies	Nephronophthisis	* 610162	*CCDC28B*	COILED-COIL DOMAIN-CONTAINING PROTEIN 28B
72	Ciliopathies	Nephronophthisis	* 604265	*CELSR2*	CADHERIN EGF LAG SEVEN-PASS G-TYPE RECEPTOR 2
73	Ciliopathies	Nephronophthisis	* 616690	*CEP104*	CENTROSOMAL PROTEIN, 104-KD
74	Ciliopathies	Nephronophthisis	* 613446	*CEP120*	CENTROSOMAL PROTEIN, 120-KD
75	Ciliopathies	Nephronophthisis	* 614848	*CEP164*	CENTROSOMAL PROTEIN, 164-KD
76	Ciliopathies	Nephronophthisis	* 610142	*CEP290*	CENTROSOMAL PROTEIN, 290-KD
77	Ciliopathies	Nephronophthisis	* 610523	*CEP41*	CENTROSOMAL PROTEIN, 41-KD
78	Ciliopathies	Nephronophthisis	* 610000	*CEP55*	CENTROSOMAL PROTEIN, 55-KD
79	Ciliopathies	Nephronophthisis	* 615847	*CEP83*	CENTROSOMAL PROTEIN, 83-KD
80	Ciliopathies	Nephronophthisis	* 614571	*CPLANE1*	CILIOGENESIS AND PLANAR POLARITY EFFECTOR 1
81	Ciliopathies	Nephronophthisis	* 611654	*CSPP1*	CENTROSOME SPINDLE POLE-ASSOCIATED PROTEIN 1
82	Ciliopathies	Nephronophthisis	* 605755	*DCDC2*	DOUBLECORTIN DOMAIN-CONTAINING PROTEIN 2
83	Ciliopathies	Nephronophthisis	* 608053	*ETFA*	ELECTRON TRANSFER FLAVOPROTEIN, ALPHA POLYPEPTIDE
84	Ciliopathies	Nephronophthisis	* 615283	*EXOC8*	EXOCYST COMPLEX COMPONENT 8
85	Ciliopathies	Nephronophthisis	* 618413	*FAM149B1*	FAMILY WITH SEQUENCE SIMILARITY 149, MEMBER B1
86	Ciliopathies	Nephronophthisis	* 613534	*FAN1*	FANCD2/FANCI-ASSOCIATED NUCLEASE 1
87	Ciliopathies	Nephronophthisis	* 610693	*HYLS1*	HYLS1 GENE
88	Ciliopathies	Nephronophthisis	* 607386	*IFT172*	INTRAFLAGELLAR TRANSPORT 172
89	Ciliopathies	Nephronophthisis	* 615870	*IFT27*	INTRAFLAGELLAR TRANSPORT 27
90	Ciliopathies	Nephronophthisis	* 608040	*IFT74*	INTRAFLAGELLAR TRANSPORT 74
91	Ciliopathies	Nephronophthisis	* 613037	*INPP5E*	INOSITOL POLYPHOSPHATE-5-PHOSPHATASE, 72-KD
92	Ciliopathies	Nephronophthisis	* 243305	*INVS*	INVERSIN
93	Ciliopathies	Nephronophthisis	* 609237	*IQCB1*	IQ MOTIF-CONTAINING PROTEIN B1
94	Ciliopathies	Nephronophthisis	* 616650	*KIAA0556*	KIAA0556 GENE
95	Ciliopathies	Nephronophthisis	* 610178	*KIAA0586*	KIAA0586 GENE
96	Ciliopathies	Nephronophthisis	* 617112	*KIAA0753*	KIAA0753 GENE
97	Ciliopathies	Nephronophthisis	* 611279	*KIF14*	KINESIN FAMILY MEMBER 14
98	Ciliopathies	Nephronophthisis	* 611254	*KIF7*	KINESIN FAMILY MEMBER 7
99	Ciliopathies	Nephronophthisis	* 606568	*LZTFL1*	LEUCINE ZIPPER TRANSCRIPTION FACTOR-LIKE 1
100	Ciliopathies	Nephronophthisis	* 616786	*MAPKBP1*	MITOGEN-ACTIVATED PROTEIN KINASE-BINDING PROTEIN 1
101	Ciliopathies	Nephronophthisis	* 604896	*MKKS*	MKKS GENE
102	Ciliopathies	Nephronophthisis	* 609883	*MKS1*	MKS1 GENE
103	Ciliopathies	Nephronophthisis	* 609799	*NEK8*	NEVER IN MITOSIS GENE A-RELATED KINASE 8
104	Ciliopathies	Nephronophthisis	* 607100	*NPHP1*	NEPHROCYSTIN 1
105	Ciliopathies	Nephronophthisis	* 608002	*NPHP3*	NEPHROCYSTIN 3
106	Ciliopathies	Nephronophthisis	* 607215	*NPHP4*	NEPHROCYSTIN 4
107	Ciliopathies	Nephronophthisis	* 602676	*PDE6D*	PHOSPHODIESTERASE 6D, cGMP-SPECIFIC, ROD, DELTA
108	Ciliopathies	Nephronophthisis	* 617835	*PDPR*	PYRUVATE DEHYDROGENASE PHOSPHATASE REGULATORY SUBUNIT
109	Ciliopathies	Nephronophthisis	* 607532	*PIBF1*	PROGESTERONE-INDUCED BLOCKING FACTOR 1

110	Ciliopathies	Nephronophthisis	* 601785	*PMM2*	PHOSPHOMANNOMUTASE 2
111	Ciliopathies	Nephronophthisis	* 614784	*POC1B*	POC1 CENTRIOLAR PROTEIN B
112	Ciliopathies	Nephronophthisis	* 610937	*RPGRIP1L*	RPGRIP1-LIKE
113	Ciliopathies	Nephronophthisis	* 613524	*SDCCAG8*	SEROLOGICALLY DEFINED COLON CANCER ANTIGEN 8
114	Ciliopathies	Nephronophthisis	* 607035	*SUFU*	SUFU NEGATIVE REGULATOR OF HEDGEHOG SIGNALING
115	Ciliopathies	Nephronophthisis	* 609863	*TCTN1*	TECTONIC FAMILY, MEMBER 1
116	Ciliopathies	Nephronophthisis	* 613846	*TCTN2*	TECTONIC FAMILY, MEMBER 2
117	Ciliopathies	Nephronophthisis	* 613847	*TCTN3*	TECTONIC FAMILY, MEMBER 3
118	Ciliopathies	Nephronophthisis	* 616183	*TMEM107*	TRANSMEMBRANE PROTEIN 107
119	Ciliopathies	Nephronophthisis	* 614459	*TMEM138*	TRANSMEMBRANE PROTEIN 138
120	Ciliopathies	Nephronophthisis	* 613277	*TMEM216*	TRANSMEMBRANE PROTEIN 216
121	Ciliopathies	Nephronophthisis	* 614949	*TMEM231*	TRANSMEMBRANE PROTEIN 231
122	Ciliopathies	Nephronophthisis	* 614423	*TMEM237*	TRANSMEMBRANE PROTEIN 237
123	Ciliopathies	Nephronophthisis	* 609884	*TMEM67*	TRANSMEMBRANE PROTEIN 67
124	Ciliopathies	Nephronophthisis	* 607380	*TRAF3IP1*	TNF RECEPTOR-ASSOCIATED FACTOR 3-INTERACTING PROTEIN 1
125	Ciliopathies	Nephronophthisis	* 610955	*TRAPPC3*	TRAFFICKING PROTEIN PARTICLE COMPLEX, SUBUNIT 3
126	Ciliopathies	Nephronophthisis	* 602290	*TRIM32*	TRIPARTITE MOTIF-CONTAINING PROTEIN 32
127	Ciliopathies	Nephronophthisis	* 612014	*TTC21B*	TETRATRICOPEPTIDE REPEAT DOMAIN-CONTAINING PROTEIN 21B
128	Ciliopathies	Nephronophthisis	* 608132	*TTC8*	TETRATRICOPEPTIDE REPEAT DOMAIN-CONTAINING PROTEIN 8
129	Ciliopathies	Nephronophthisis	* 617778	*TXNDC15*	THIOREDOXIN DOMAIN-CONTAINING PROTEIN 15
130	Ciliopathies	Nephronophthisis	* 613580	*WDPCP*	WD REPEAT-CONTAINING PLANAR CELL POLARITY EFFECTOR
131	Ciliopathies	Nephronophthisis	* 608151	*WDR19*	WD REPEAT-CONTAINING PROTEIN 19
132	Ciliopathies	Nephronophthisis	* 613602	*WDR35*	WD REPEAT-CONTAINING PROTEIN 35
133	Ciliopathies	Nephronophthisis	* 613553	*XPNPEP3*	X-PROLYL AMINOPEPTIDASE 3
134	Ciliopathies	Nephronophthisis	* 604557	*ZNF423*	ZINC FINGER PROTEIN 423
135	CAKUT	CAKUT	* 179820	*ACE*	ANGIOTENSIN I-CONVERTING ENZYME
136	CAKUT	CAKUT	+ 106180	*AGT*	ANGIOTENSINOGEN
137	CAKUT	CAKUT	* 106165	*AGTR1*	ANGIOTENSIN RECEPTOR 1
138	CAKUT	CAKUT	* 605558	*FGF20*	FIBROBLAST GROWTH FACTOR 20
139	CAKUT	CAKUT	* 608296	*FIBP*	FIBROBLAST GROWTH FACTOR, ACIDIC, INTRACELLULAR BINDING PROTEIN
140	CAKUT	CAKUT	* 608945	*FREM2*	FRAS1-RELATED EXTRACELLULAR MATRIX PROTEIN 2
141	CAKUT	CAKUT	* 604597	*GRIP1*	GLUTAMATE RECEPTOR-INTERACTING PROTEIN 1
142	CAKUT	CAKUT	* 604063	*ITGA8*	INTEGRIN, ALPHA-8
143	CAKUT	CAKUT	* 604270	*LRP4*	LOW DENSITY LIPOPROTEIN RECEPTOR-RELATED PROTEIN 4
144	CAKUT	CAKUT	* 179820	*REN*	RENIN
145	CAKUT	CAKUT	* 118494	*CHRM3*	CHOLINERGIC RECEPTOR, MUSCARINIC, 3
146	CAKUT	CAKUT	* 607830	*FRAS1*	FRASER EXTRACELLULAR MATRIX COMPLEX SUBUNIT 1
147	CAKUT	CAKUT	* 608944	*FREM1*	FRAS1-RELATED EXTRACELLULAR MATRIX PROTEIN 1
148	CAKUT	CAKUT	* 613469	*HPSE2*	HEPARANASE 2

149	CAKUT	CAKUT	* 608869	*LRIG2*	LEUCINE-RICH REPEATS- AND IMMUNOGLOBULIN-LIKE DOMAINS-CONTAINING PROTEIN 2

Abbreviations: AS, Alport syndrome; CAKUT, congenital anomalies of the kidney and urinary tract; FSGS, focal segmental glomerulosclerosis; SRNS, steroid-resistant nephrotic syndrome; TBMN, thin basement membrane nephropathy; OMIM, Online Mendelian Inheritance in Man.

Supplementary Table 2. List of included variants and their allele frequencies in our in-house and gnomAD database.

https://doi.org/10.6084/m9.figshare.21972917

Supplementary Table 3. Detailed list of excluded variants.

https://doi.org/10.6084/m9.figshare.21972986

Supplementary Table 4. List of all lifetime risks per 100,000 individuals for all 149 autosomal recessive kidney disease genes.

Gene	Number of disease-causing variants in gnomAD dataset	Number of disease-causing alleles in European (non-Finnish) gnomAD	Number of disease-causing alleles in worldwide gnomAD	Number of disease-causing variants in-house database	Number of disease-causing alleles in-house database	European (non-Finnish) gnomAD			Worldwide gnomAD			In-house		
						Lifetime risk	Lifetime risk lower CI	Lifetime risk upper CI	Lifetime risk	Lifetime risk lower CI	Lifetime risk upper CI	Lifetime risk	Lifetime risk lower CI	Lifetime risk upper CI
ARHGDIA	10	4	24	1	1	0.0004	0.0000	0.0034	0.0018	0.0006	0.0044	0.0000	0.0000	0.0018
COL4A3	275	316	833	63	158	1.2974	1.0010	1.6667	1.7277	1.4754	2.0164	1.1141	0.7683	1.5863
COL4A4	247	268	638	50	114	0.8542	0.6440	1.1211	0.9210	0.7686	1.0990	0.5784	0.3721	0.8764
COQ2	23	22	85	8	14	0.0087	0.0030	0.0221	0.0380	0.0227	0.0615	0.0102	0.0025	0.0326
COQ6	42	38	83	3	3	0.0251	0.0114	0.0513	0.0212	0.0126	0.0346	0.0004	0.0000	0.0047
COQ8B	49	53	133	14	26	0.0254	0.0131	0.0465	0.0365	0.0243	0.0537	0.0351	0.0132	0.0830
CRB2	67	91	165	15	83	0.9192	0.5595	1.4624	0.4484	0.3117	0.6338	0.3588	0.2130	0.5835
DAAM2	6	5	14	6	8	0.0003	0.0000	0.0023	0.0007	0.0002	0.0022	0.0033	0.0005	0.0150
DGKE	27	42	64	8	21	0.0206	0.0097	0.0406	0.0127	0.0070	0.0220	0.0229	0.0076	0.0594
EMP2	12	15	28	3	4	0.0032	0.0008	0.0099	0.0048	0.0019	0.0110	0.0008	0.0000	0.0066
ITGA3	39	22	68	5	10	0.0104	0.0036	0.0264	0.0138	0.0077	0.0236	0.0052	0.0009	0.0202
KANK1	75	50	167	17	29	0.0533	0.0269	0.0995	0.4628	0.3225	0.6528	0.0436	0.0173	0.0984
KANK2	15	24	113	3	4	0.0047	0.0017	0.0114	0.0188	0.0121	0.0285	0.0008	0.0000	0.0066
KANK4	38	26	114	9	11	0.0082	0.0031	0.0195	0.0313	0.0201	0.0474	0.0063	0.0013	0.0230
LAMB2	93	94	168	16	22	0.1218	0.0747	0.1924	0.0722	0.0503	0.1017	0.0246	0.0084	0.0626
MYO1E	36	18	40	4	5	0.0156	0.0047	0.0435	0.0120	0.0056	0.0242	0.0013	0.0001	0.0083
NPHS1	90	193	619	20	57	0.4314	0.3086	0.5942	0.7159	0.5957	0.8565	0.1473	0.0779	0.2645
NPHS2	53	276	417	23	115	0.6016	0.4554	0.7865	0.2900	0.2316	0.3608	0.5781	0.3727	0.8744
NUP107	58	51	92	4	8	0.0379	0.0192	0.0703	0.0205	0.0125	0.0326	0.0033	0.0005	0.0147
NUP133	40	26	62	6	9	0.0262	0.0098	0.0618	0.0172	0.0093	0.0301	0.0041	0.0007	0.0172
NUP160	58	50	119	3	5	0.0684	0.0345	0.1277	0.0466	0.0303	0.0700	0.0013	0.0001	0.0083
NUP205	31	20	39	3	3	0.0118	0.0038	0.0312	0.0087	0.0040	0.0176	0.0005	0.0000	0.0048
NUP85	5	3	7	2	2	0.0001	0.0000	0.0007	0.0001	0.0000	0.0004	0.0002	0.0000	0.0034
NUP93	33	21	44	4	12	0.0093	0.0031	0.0240	0.0069	0.0033	0.0134	0.0073	0.0016	0.0255
PDSS2	22	15	43	4	7	0.0017	0.0004	0.0051	0.0081	0.0038	0.0158	0.0025	0.0003	0.0124
PLCE1	65	43	129	9	11	0.0189	0.0090	0.0370	0.0426	0.0282	0.0630	0.0062	0.0012	0.0226
PTPRO	31	23	58	5	6	0.0151	0.0053	0.0376	0.0062	0.0033	0.0110	0.0018	0.0002	0.0101
SGPL1	18	10	22	2	2	0.0016	0.0003	0.0062	0.0013	0.0005	0.0034	0.0002	0.0000	0.0033

SMARCAL1	36	84	149	16	47	0.0530	0.0316	0.0860	0.0395	0.0269	0.0569	0.1039	0.0512	0.1976
TRIM8	5	3	5	2	2	0.0001	0.0000	0.0009	0.0001	0.0000	0.0003	0.0002	0.0000	0.0033
WDR73	28	36	100	10	19	0.0242	0.0107	0.0504	0.0252	0.0157	0.0392	0.0179	0.0056	0.0486
BSND	22	20	125	6	32	0.0061	0.0020	0.0162	0.0237	0.0155	0.0352	0.0447	0.0187	0.0972
CLCNKA	67	397	758	5	7	1.6395	1.3019	2.0502	1.0184	0.8629	1.1976	0.0024	0.0003	0.0121
CLCNKB	66	110	285	18	31	0.6309	0.4025	0.9630	1.2170	0.9257	1.5841	0.0466	0.0192	0.1025
CLDN16	17	28	59	7	25	0.0051	0.0020	0.0116	0.0118	0.0063	0.0210	0.0268	0.0098	0.0643
CLDN19	10	5	11	5	6	0.0015	0.0001	0.0095	0.0008	0.0002	0.0028	0.0018	0.0002	0.0099
CNNM2	6	3	6	2	2	0.0001	0.0000	0.0008	0.0001	0.0000	0.0003	0.0002	0.0000	0.0032
EGF	52	33	142	6	8	0.0113	0.0048	0.0243	0.0498	0.0336	0.0723	0.0031	0.0004	0.0142
KCNJ1	20	63	221	8	36	0.0392	0.0214	0.0684	0.0923	0.0675	0.1245	0.0581	0.0256	0.1209
SLC12A1	68	65	158	8	14	0.0646	0.0356	0.1118	0.0759	0.0523	0.1081	0.0095	0.0024	0.0303
TRPM6	52	40	73	10	17	0.0157	0.0072	0.0314	0.0135	0.0077	0.0226	0.0140	0.0040	0.0403
SLC12A3	150	567	1004	52	184	2.2753	1.8777	2.7435	1.4788	1.2810	1.7024	1.4758	1.0472	2.0479
ATP6V0A4	66	55	150	15	30	0.0405	0.0211	0.0735	0.0445	0.0303	0.0639	0.0420	0.0170	0.0937
ATP6V1B1	35	23	59	3	4	0.0039	0.0014	0.0096	0.0080	0.0043	0.0142	0.0008	0.0000	0.0062
BCS1L	61	247	469	22	55	2.8444	2.1192	3.7742	0.3071	0.2484	0.3773	0.1316	0.0687	0.2388
CA2	21	7	23	3	4	0.0034	0.0004	0.0169	0.0026	0.0009	0.0064	0.0008	0.0000	0.0062
SLC4A4	19	22	29	5	8	0.1931	0.0659	0.4900	0.0367	0.0146	0.0828	0.0029	0.0004	0.0131
DZIP1L	64	54	130	5	9	0.0265	0.0138	0.0484	0.0473	0.0313	0.0698	0.0039	0.0006	0.0164
PKHD1	221	393	1310	46	181	2.2706	1.8009	2.8423	2.8933	2.5524	3.2727	1.2839	0.8422	1.9123
AHI1	75	126	389	11	24	0.1763	0.1160	0.2619	0.3210	0.2542	0.4024	0.0280	0.0100	0.0683
ALMS1	161	168	321	44	83	0.4122	0.2876	0.5809	0.2416	0.1868	0.3099	0.3344	0.1985	0.5438
ANKS6	30	34	71	8	12	0.0180	0.0077	0.0383	0.0128	0.0072	0.0216	0.0070	0.0015	0.0243
ARL13B	30	16	53	4	5	0.0037	0.0010	0.0110	0.0069	0.0036	0.0127	0.0012	0.0001	0.0079
ARL3	6	7	8	0	0	0.0088	0.0011	0.0437	0.0017	0.0002	0.0076	-	-	-
ARL6	19	14	41	6	10	0.0031	0.0008	0.0099	0.0084	0.0039	0.0166	0.0046	0.0006	0.0231
ARMC9	52	72	120	7	13	0.0416	0.0237	0.0700	0.0429	0.0279	0.0643	0.0082	0.0019	0.0272
B9D1	20	32	71	7	7	0.0111	0.0046	0.0241	0.0149	0.0084	0.0252	0.0024	0.0003	0.0118
B9D2	10	17	20	3	4	0.0449	0.0129	0.1290	0.0007	0.0002	0.0018	0.0008	0.0000	0.0062
BBIP1	10	11	19	1	2	0.0133	0.0027	0.0487	0.0064	0.0020	0.0174	0.0002	0.0000	0.0032
BBS1	47	41	95	9	13	0.0576	0.0269	0.1146	0.0384	0.0236	0.0606	0.0082	0.0019	0.0272
BBS10	57	204	304	11	46	0.2829	0.2043	0.3863	0.1504	0.1154	0.1942	0.1027	0.0502	0.1968
BBS12	47	98	123	8	29	0.0751	0.0465	0.1175	0.0245	0.0160	0.0365	0.0408	0.0162	0.0922
BBS2	62	126	238	15	27	0.1330	0.0875	0.1976	0.1024	0.0758	0.1367	0.0340	0.0130	0.0791
BBS4	53	66	309	12	54	0.0388	0.0215	0.0668	0.2452	0.1885	0.3159	0.1279	0.0398	0.3475
BBS5	28	35	64	2	15	0.0094	0.0041	0.0198	0.0074	0.0043	0.0136	0.0157	0.0041	0.0482
BBS7	55	80	156	11	43	0.0511	0.0300	0.0839	0.0382	0.0263	0.0546	0.0898	0.0427	0.1758
BBS9	68	76	136	13	29	0.1207	0.0699	0.2006	0.0753	0.0503	0.1102	0.0408	0.0162	0.0922

C2CD3	79	123	179	10	26	0.1928	0.1262	0.2878	0.0969	0.0684	0.1351	0.0328	0.0123	0.0775
C8orf37	14	13	20	4	4	0.0053	0.0012	0.0175	0.0019	0.0006	0.0051	0.0008	0.0000	0.0061
CC2D2A	115	147	402	26	63	0.3399	0.2311	0.4904	0.4853	0.3858	0.6061	0.1910	0.1044	0.3334
CCDC28B	17	17	31	4	6	0.0025	0.0007	0.0073	0.0029	0.0012	0.0065	0.0017	0.0002	0.0097
CELSR2	31	22	33	7	9	0.0083	0.0028	0.0211	0.0056	0.0024	0.0120	0.0039	0.0006	0.0163
CEP104	57	102	152	10	14	0.1111	0.0696	0.1724	0.0495	0.0339	0.0710	0.0094	0.0023	0.0300
CEP120	59	33	86	8	10	0.0171	0.0073	0.0368	0.0434	0.0260	0.0700	0.0048	0.0009	0.0187
CEP164	79	65	162	18	25	0.0948	0.0523	0.1641	0.0986	0.0683	0.1398	0.0301	0.0111	0.0722
CEP290	202	324	625	56	111	1.7418	1.3484	2.2305	1.2212	1.0172	1.4597	0.5929	0.3791	0.9033
CEP41	3	2	10	3	3	0.0000	0.0000	0.0005	0.0002	0.0000	0.0006	0.0004	0.0000	0.0046
CEP55	33	73	140	7	28	0.0776	0.0444	0.1303	0.0418	0.0281	0.0609	0.0377	0.0147	0.0864
CEP83	47	65	108	6	16	0.0292	0.0161	0.0506	0.0089	0.0056	0.0136	0.0123	0.0034	0.0365
CPLANE1	150	256	455	39	130	1.0920	0.8178	1.4422	0.8358	0.6740	1.0301	0.8130	0.5388	1.2001
CSPP1	98	142	282	17	33	0.2314	0.1562	0.3360	0.1690	0.1284	0.2204	0.0524	0.0222	0.1126
DCDC2	35	30	59	8	19	0.0071	0.0029	0.0158	0.0077	0.0041	0.0136	0.0174	0.0054	0.0472
ETFA	29	40	64	3	4	0.0209	0.0097	0.0420	0.0164	0.0090	0.0284	0.0008	0.0000	0.0061
EXOC8	0	0	0	3	4							0.0008	0.0000	0.0061
FAM149B1	52	39	149	3	4	0.0382	0.0174	0.0772	0.0975	0.0664	0.1403	0.0008	0.0000	0.0061
FAN1	101	191	393	20	47	0.3379	0.2413	0.4662	0.3433	0.2722	0.4299	0.1063	0.0524	0.2022
HYLS1	19	107	399	6	21	0.0703	0.0446	0.1080	0.2114	0.1679	0.2643	0.0195	0.0065	0.0506
IFT172	122	79	182	18	33	0.1332	0.0780	0.2193	0.1084	0.0767	0.1507	0.0524	0.0222	0.1126
IFT27	14	36	47	1	7	0.0200	0.0088	0.0417	0.0068	0.0033	0.0129	0.0023	0.0003	0.0116
IFT74	39	55	98	12	28	0.0207	0.0108	0.0375	0.0173	0.0107	0.0270	0.0375	0.0147	0.0860
INPP5E	41	49	99	13	22	0.0595	0.0298	0.1117	0.0356	0.0221	0.0556	0.0220	0.0075	0.0559
INVS	71	80	154	14	29	0.0687	0.0404	0.1128	0.0753	0.0517	0.1078	0.0401	0.0160	0.0906
IQCB1	54	127	200	10	33	0.2535	0.2398	0.5396	0.1038	0.0747	0.1422	0.0520	0.0220	0.1117
KIAA0556	106	207	390	20	75	0.3785	0.2740	0.5157	0.3344	0.2649	0.4190	0.2684	0.1548	0.4475
KIAA0586	73	110	208	29	50	0.1135	0.0724	0.1733	0.1233	0.0893	0.1679	0.1193	0.0602	0.2227
KIAA0753	70	100	181	9	23	0.1259	0.0785	0.1962	0.0723	0.0511	0.1007	0.0252	0.0088	0.0628
KIF14	64	55	102	10	25	0.0434	0.0227	0.0788	0.0393	0.0246	0.0610	0.0298	0.0110	0.0716
KIF7	83	74	144	23	33	0.0975	0.0560	0.1632	0.0917	0.0620	0.1328	0.0519	0.0220	0.1116
LZTFL1	14	5	18	1	1	0.0024	0.0002	0.0157	0.0024	0.0007	0.0066	0.0000	0.0000	0.0019
MAPKBP1	33	14	38	3	3	0.0015	0.0004	0.0048	0.0032	0.0014	0.0065	0.0004	0.0000	0.0045
MKKS	40	43	93	12	19	0.0351	0.0167	0.0687	0.0276	0.0169	0.0437	0.0167	0.0052	0.0453
MKS1	50	43	125	10	21	0.0238	0.0113	0.0466	0.0302	0.0199	0.0450	0.0210	0.0070	0.0545
NEK8	45	38	100	8	17	0.0142	0.0064	0.0290	0.0171	0.0107	0.0267	0.0138	0.0040	0.0396
NPHP1	54	58	139	9	13	1.1667	0.6207	2.0829	0.5920	0.3978	0.8628	0.0081	0.0019	0.0268
NPHP3	86	159	286	17	40	0.3375	0.2330	0.4801	0.1820	0.1385	0.2369	0.0760	0.0351	0.1525
NPHP4	113	88	216	17	31	0.0906	0.0546	0.1454	0.1794	0.1308	0.2429	0.0458	0.0189	0.1009

PDE6D	8	5	10	0	0	0.0002	0.0000	0.0013	0.0002	0.0000	0.0006	-	-	-
PDPR	45	96	178	8	38	0.6823	0.4211	1.0727	0.3816	0.2691	0.5325	0.0686	0.0310	0.1401
PIBF1	58	73	137	7	11	0.0996	0.0570	0.1673	0.0685	0.0459	0.1002	0.0058	0.0012	0.0212
PMM2	72	263	509	19	75	0.6560	0.4932	0.8632	0.4850	0.3958	0.5910	0.2294	0.1323	0.3826
POC1B	26	28	63	7	16	0.0059	0.0023	0.0134	0.0080	0.0043	0.0139	0.0122	0.0034	0.0361
RPGRIP1L	124	123	257	15	30	0.2084	0.1364	0.3111	0.1813	0.1358	0.2393	0.0426	0.0172	0.0949
SDCCAG8	59	55	130	8	11	0.0480	0.0251	0.0871	0.0560	0.0371	0.0827	0.0057	0.0012	0.0210
SUFU	2	1	3	1	1	0.0004	0.0000	0.0171	0.0005	0.0000	0.0048	0.0000	0.0000	0.0019
TCTN1	48	59	110	6	10	0.0326	0.0174	0.0580	0.0217	0.0138	0.0331	0.0047	0.0009	0.0184
TCTN2	54	49	96	7	11	0.0709	0.0355	0.1332	0.0417	0.0257	0.0656	0.0057	0.0012	0.0210
TCTN3	40	49	94	9	20	0.0719	0.0360	0.1351	0.0388	0.0238	0.0612	0.0189	0.0061	0.0502
TMEM107	14	6	19	2	2	0.0003	0.0000	0.0015	0.0028	0.0009	0.0077	0.0002	0.0000	0.0031
TMEM138	12	11	24	5	9	0.0044	0.0009	0.0160	0.0203	0.0073	0.0497	0.0035	0.0006	0.0145
TMEM216	11	27	45	3	7	0.0085	0.0032	0.0197	0.0039	0.0019	0.0076	0.0023	0.0003	0.0115
TMEM231	23	68	102	8	23	0.0832	0.0466	0.1424	0.0289	0.0181	0.0448	0.0250	0.0088	0.0623
TMEM237	25	46	65	4	11	0.0196	0.0096	0.0375	0.0076	0.0042	0.0132	0.0057	0.0012	0.0210
TMEM67	90	164	266	16	31	0.2256	0.1567	0.3193	0.1444	0.1087	0.1897	0.0452	0.0186	0.0996
TRAF3IP1	39	28	66	6	7	0.0432	0.0169	0.0991	0.0323	0.0179	0.0557	0.0023	0.0003	0.0115
TRAPPC3	2	1	2	0	0	0.0000	0.0000	0.0003	0.0000	0.0000	0.0001	-	-	-
TRIM32	30	24	57	3	3	0.0324	0.0116	0.0791	0.0179	0.0094	0.0321	0.0004	0.0000	0.0045
TTC21B	92	118	227	14	23	0.1323	0.0858	0.1992	0.1338	0.0984	0.1799	0.0250	0.0088	0.0623
TTC8	28	18	34	5	7	0.0050	0.0015	0.0140	0.0049	0.0021	0.0104	0.0023	0.0003	0.0115
TXNDC15	14	48	62	5	11	0.0297	0.0147	0.0561	0.0113	0.0062	0.0199	0.0057	0.0012	0.0210
WDPCP	48	36	74	6	10	0.0460	0.0203	0.0957	0.0229	0.0132	0.0383	0.0047	0.0009	0.0184
WDR19	62	58	116	6	10	0.0500	0.0266	0.0893	0.0509	0.0329	0.0768	0.0047	0.0009	0.0184
WDR35	92	131	276	10	47	0.2377	0.1577	0.3504	0.2182	0.1652	0.2853	0.1040	0.0512	0.1978
XPNPEP3	26	36	87	2	2	0.0134	0.0059	0.0278	0.0164	0.0098	0.0264	0.0002	0.0000	0.0031
ZNF423	4	4	5	0	0	0.0001	0.0000	0.0009	0.0000	0.0000	0.0002	-	-	-
ACE	96	139	320	16	26	0.1992	0.1339	0.2904	0.1984	0.1532	0.2545	0.0318	0.0119	0.0751
AGT	19	7	51	4	4	0.0013	0.0002	0.0067	0.0229	0.0116	0.0424	0.0008	0.0000	0.0060
AGTR1	24	13	44	2	2	0.0028	0.0007	0.0093	0.0090	0.0043	0.0175	0.0002	0.0000	0.0031
FGF20	5	4	9	4	7	0.0025	0.0001	0.0195	0.0046	0.0008	0.0192	0.0023	0.0003	0.0115
FIBP	21	15	28	3	4	0.0038	0.0010	0.0117	0.0039	0.0015	0.0088	0.0008	0.0000	0.0060
FREM2	90	81	135	6	9	0.1647	0.0971	0.2695	0.1004	0.0670	0.1471	0.0038	0.0006	0.0159
GRIP1	25	21	51	1	1	0.0293	0.0097	0.0760	0.0218	0.0111	0.0404	0.0000	0.0000	0.0019
ITGA8	51	30	87	4	9	0.0110	0.0045	0.0246	0.0147	0.0089	0.0237	0.0038	0.0006	0.0159
LRP4	44	31	56	5	6	0.0110	0.0045	0.0242	0.0097	0.0051	0.0175	0.0017	0.0002	0.0095
REN	28	28	70	4	8	0.0085	0.0033	0.0194	0.0087	0.0049	0.0148	0.0030	0.0004	0.0136
CHRM3	3	3	4	1	4	0.0001	0.0000	0.0008	0.0000	0.0000	0.0002	0.0006	0.0000	0.0048

FRAS1	171	163	290	27	41	0.3306	0.2293	0.4683	0.2461	0.1876	0.3196	0.0646	0.0302	0.1286
FREM1	136	96	254	19	29	0.1125	0.0694	0.1769	0.1723	0.1289	0.2279	0.0317	0.0126	0.0717
HPSE2	26	67	116	9	23	0.0294	0.0164	0.0504	0.0274	0.0177	0.0414	0.0199	0.0070	0.0496
LRIG2	76	93	153	6	10	0.1293	0.0791	0.2048	0.0751	0.0515	0.1076	0.0038	0.0007	0.0148

In empty cells ("-") no lifetime risk could be determined due to the absence of disease-causing variants in the datasets (in-house or gnomAD). Abbreviations: CI, confidence interval.

Supplementary Figures

Supplementary Figure 1. Flowchart of the analysis.

The lifetime risk of an autosomal recessive kidney disease is defined as the proportion of a population that will develop the kidney disease at some point in life. After a literature review, we identified 149 genes (*i*) associated with an autosomal recessive kidney disease. The three publicly available databases, ClinVar, Human Gene Mutation Database (HGMD), and Leiden Open Variation Database (LOVD), were queried for (likely) pathogenic variants. Additionally, our in-house database was searched for loss-of-function variants not described in one of the databases. Afterwards, variants were rated towards their pathogenicity according to the American College of Medical Genetics and Genomics (ACMG) recommendations and current amendments. In a defined set of 12,912 (likely) pathogenic variants (n_j), allele frequencies (q_{ij}) were collected in our in-house database as well as in gnomAD. Finally, according to the Hardy-Weinberg equilibrium ($p^2 + 2pq + q^2 = 1$) summation of allele frequencies (q_{ij}) was squared to determine the lifetime risk per gene (R_i). The total sum of lifetime risks per gene resulted in the overall lifetime risk (R_{total}) for an autosomal recessive kidney disease.

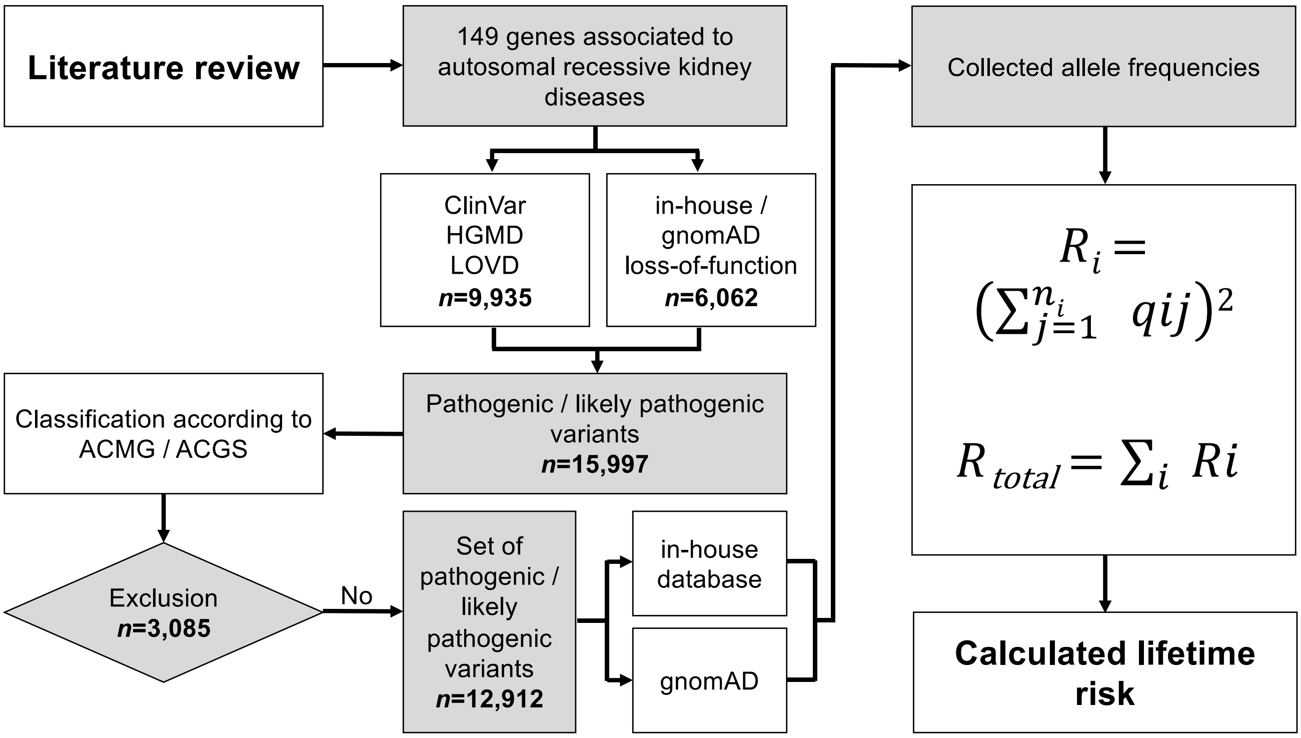

Supplementary Figure 2. Calculated lifetime risk of genes associated with autosomal recessive glomerulopathies.

Comparison of the lifetime risks per 100,000 of different monogenic kidney diseases according to the gnomAD and in-house datasets calculated independently for the European (non-Finnish) and worldwide dataset. Error bars represent 95% confidence intervals.

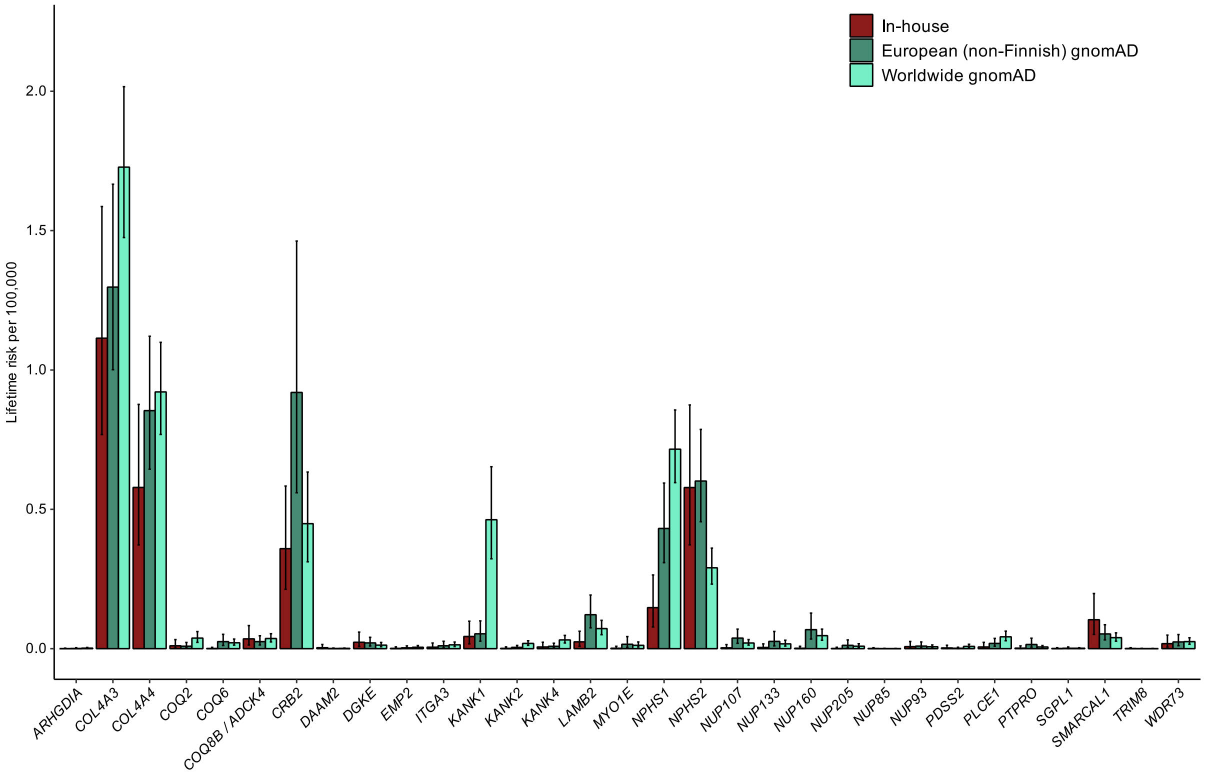

Supplementary Figure 3. Calculated lifetime risk of genes associated with autosomal recessive tubulopathies.

Comparison of the lifetime risks per 100,000 of different monogenic kidney diseases according to the gnomAD and in-house datasets calculated independently for the European (non-Finnish) and worldwide dataset. Error bars represent 95% confidence intervals.

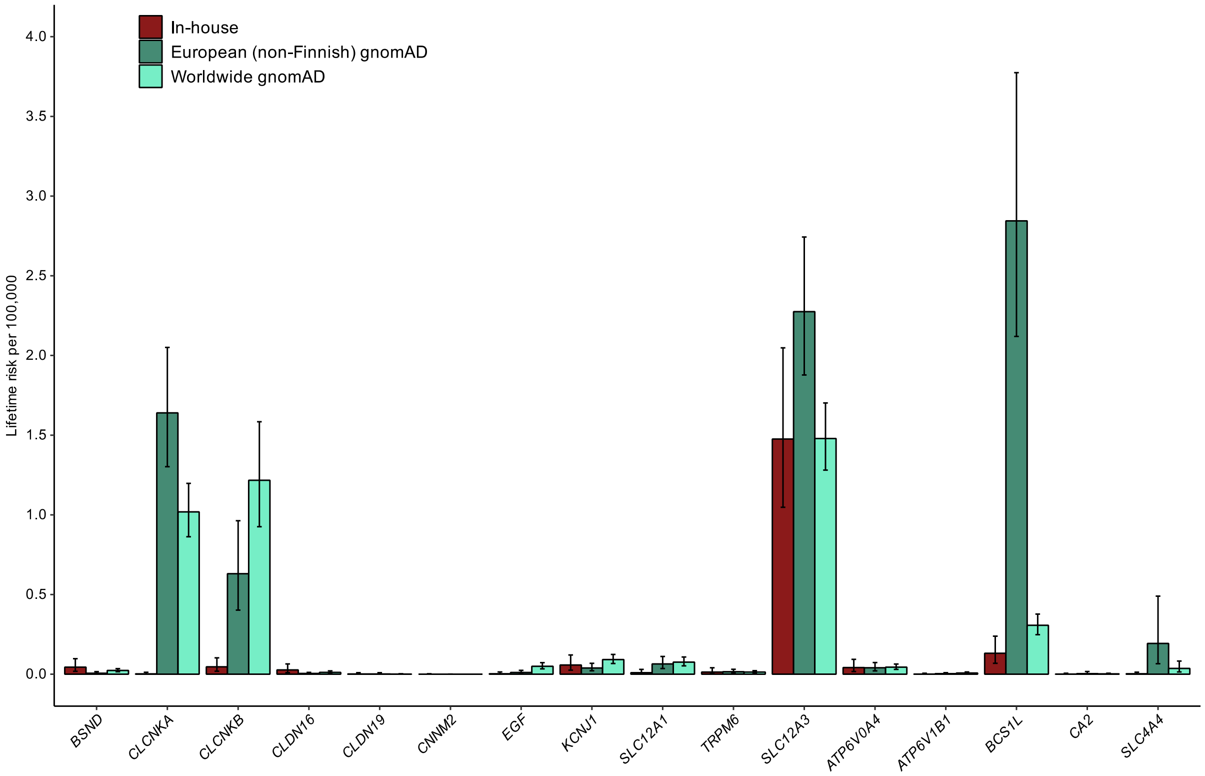

Supplementary Figure 4. Calculated lifetime risk of genes associated with autosomal recessive ciliopathies.

Comparison of the lifetime risks per 100,000 of different monogenic kidney diseases according to the gnomAD and in-house datasets calculated independently for the European (non-Finnish) and worldwide dataset. Error bars represent 95% confidence intervals.

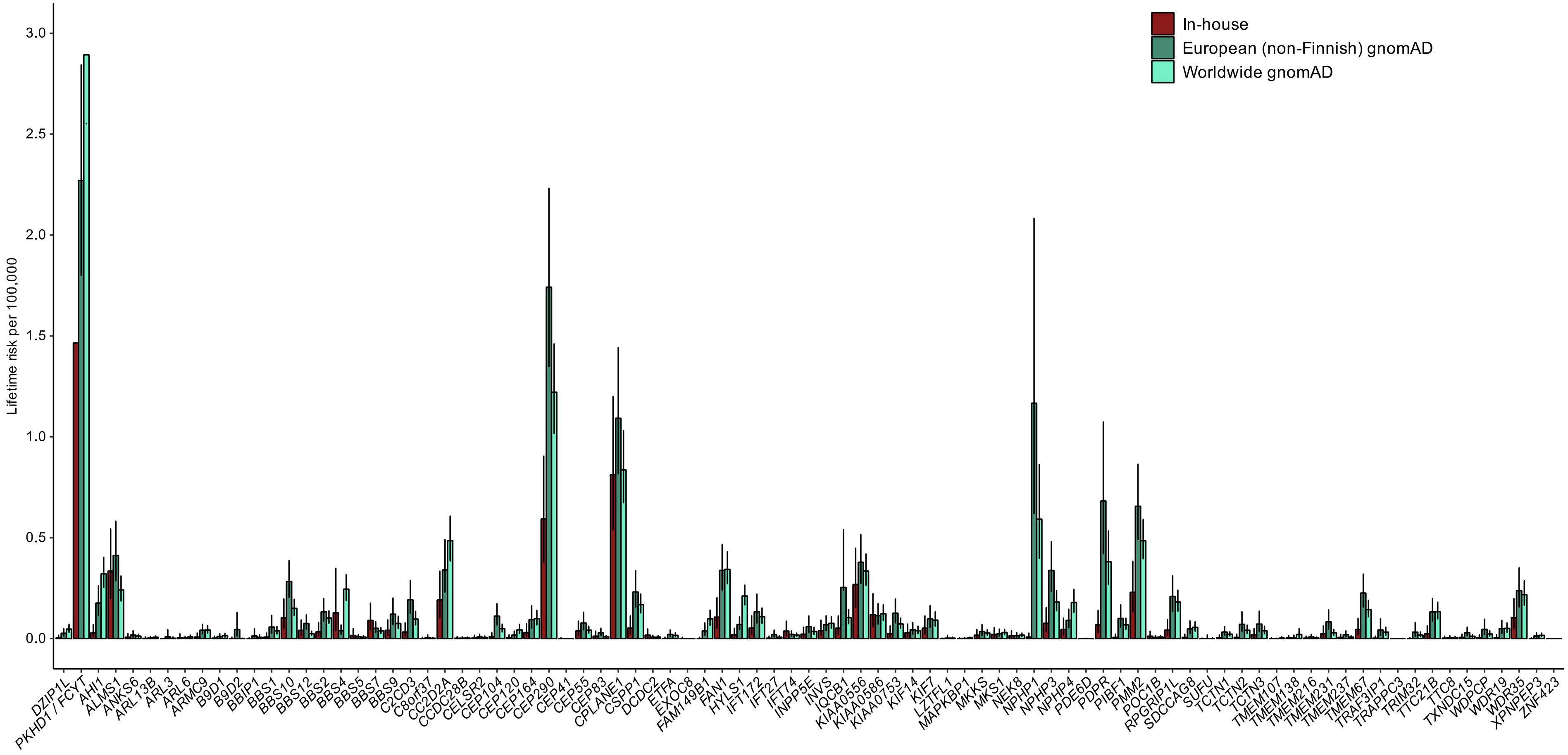

Supplementary Figure 5. Calculated lifetime risk of genes associated with autosomal recessive CAKUT.

Comparison of the lifetime risks per 100,000 of different monogenic kidney diseases according to the gnomAD and in-house datasets calculated independently for the European (non-Finnish) and worldwide dataset. Error bars represent 95% confidence intervals.

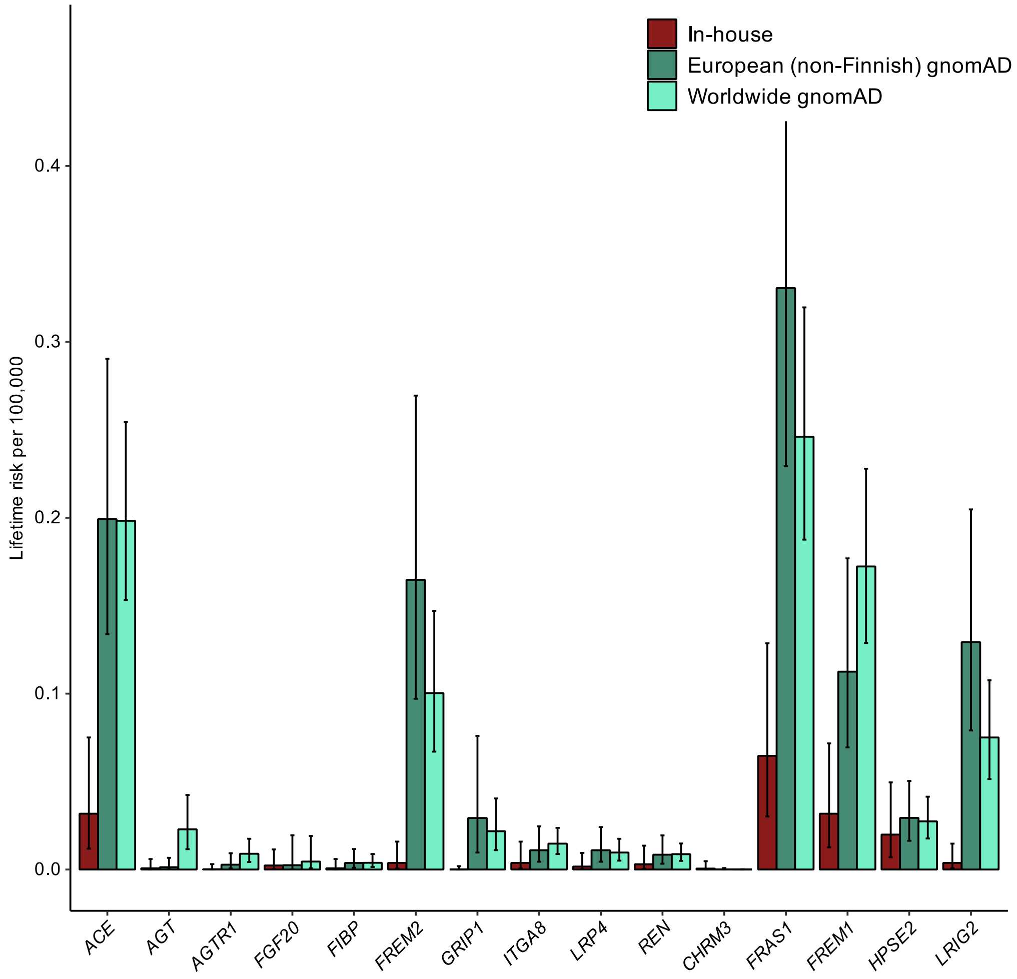

Checklist of information that should be included in new reports of global health estimates

Item #	Checklist item	Reported on page #
**Objectives and funding**		
**1**	Define the indicator(s), populations (including age, sex, and geographic entities), and time period(s) for which estimates were made.	4ff
**2**	List the funding sources for the work.	15
**Data Inputs**		
For all data inputs from multiple sources that are synthesized as part of the study:		
**3**	Describe how the data were identified and how the data were accessed.	4ff
**4**	Specify the inclusion and exclusion criteria. Identify all ad-hoc exclusions.	4ff
**5**	Provide information on all included data sources and their main characteristics. For each data source used, report reference information or contact name/institution, population represented, data collection method, year(s) of data collection, sex and age range, diagnostic criteria or measurement method, and sample size, as relevant.	4ff
**6**	Identify and describe any categories of input data that have potentially important biases (e.g., based on characteristics listed in item 5).	4ff
For data inputs that contribute to the analysis but were not synthesized as part of the study:		
**7**	Describe and give sources for any other data inputs.	4ff
For all data inputs:		
**8**	Provide all data inputs in a file format from which data can be efficiently extracted (e.g., a spreadsheet rather than a PDF), including all relevant meta-data listed in item 5. For any data inputs that cannot be shared because of ethical or legal reasons, such as third-party ownership, provide a contact name or the name of the institution that retains the right to the data.	15, Supplementary Material
**Data analysis**		
**9**	Provide a conceptual overview of the data analysis method. A diagram may be helpful.	4f, Supplementary Figure 1

**10**	Provide a detailed description of all steps of the analysis, including mathematical formulae. This description should cover, as relevant, data cleaning, data pre-processing, data adjustments and weighting of data sources, and mathematical or statistical model(s).	4ff
**11**	Describe how candidate models were evaluated and how the final model(s) were selected.	4ff
**12**	Provide the results of an evaluation of model performance, if done, as well as the results of any relevant sensitivity analysis.	n.a.
**13**	Describe methods for calculating uncertainty of the estimates. State which sources of uncertainty were, and were not, accounted for in the uncertainty analysis.	6, 13
**14**	State how analytic or statistical source code used to generate estimates can be accessed.	6
**Results and Discussion**		
**15**	Provide published estimates in a file format from which data can be efficiently extracted.	15
**16**	Report a quantitative measure of the uncertainty of the estimates (e.g. uncertainty intervals).	7ff
**17**	Interpret results in light of existing evidence. If updating a previous set of estimates, describe the reasons for changes in estimates.	10ff
**18**	Discuss limitations of the estimates. Include a discussion of any modelling assumptions or data limitations that affect interpretation of the estimates.	13

This checklist should be used in conjunction with the GATHER statement and Explanation and Elaboration document, found on gather-statement.org

Supplementary References

S1. Riedhammer KM, Ćomić J, Tasic V, *et al.* Exome sequencing in individuals with congenital anomalies of the kidney and urinary tract (CAKUT): a single-center experience. *European journal of human genetics : EJHG* 2023.

S2. Kremer LS, Bader DM, Mertes C, *et al.* Genetic diagnosis of Mendelian disorders via RNA sequencing. *Nat Commun* 2017; **8:** 15824.

S3. DePristo MA, Banks E, Poplin R, *et al.* A framework for variation discovery and genotyping using next-generation DNA sequencing data. *Nat Genet* 2011; **43:** 491-498.

S4. Van der Auwera GA, Carneiro MO, Hartl C, *et al.* From FastQ data to high confidence variant calls: the Genome Analysis Toolkit best practices pipeline. *Curr Protoc Bioinformatics* 2013; **43:** 11.10.11-11.10.33.

S5. Ye K, Schulz MH, Long Q, *et al.* Pindel: a pattern growth approach to detect break points of large deletions and medium sized insertions from paired-end short reads. *Bioinformatics (Oxford, England)* 2009; **25:** 2865-2871.

S6. Plagnol V, Curtis J, Epstein M, *et al.* A robust model for read count data in exome sequencing experiments and implications for copy number variant calling. *Bioinformatics* 2012; **28:** 2747-2754.
